# Social capital insights from Healthy Settings needs assessment in Malawi

**DOI:** 10.1371/journal.pone.0206156

**Published:** 2018-10-19

**Authors:** Sarah Rippon, Tara K. Beattie, Kingsley Lungu, Save Kumwenda, Tracy Morse

**Affiliations:** 1 Department of Civil and Environmental Engineering, University of Strathclyde, Glasgow, Scotland; 2 Department of Environmental Health, University of Malawi – The Polytechnic, Blantyre, Malawi; 3 Centre for Water, Sanitation, Hygiene and Appropriate Technology Development, University of Malawi – The Polytechnic, Blantyre, Malawi; Sefako Makgatho Health Sciences University, SOUTH AFRICA

## Abstract

Despite global health improvements, substantial challenges in social determinants of health and poverty remain in rural communities in low-income countries. Public health theorists suggest that communities with high social capital are less vulnerable to such challenges and more likely to participate in community development. This research examines levels of social capital amongst rural communities in southern Malawi through data gathered as part of a participatory needs assessment for a Healthy Settings project, and discusses the potential benefits of having access to such data before project implementation. Social capital data was collected during 108 focus group discussions in 18 communities (split by gender, age, status) by adapting an existing mixed methods measurement tool, the Schutte tool. Five indicators were measured: sense of belonging, friendship, reliance, ability to work together and influence. Mean results showed all 18 communities had medium-high levels of social capital. Means from each group in the 18 communities highlighted the lowest social capital among the youth groups and the highest with the leaders. A more detailed breakdown highlighted that all groups had a strong sense of belonging to the community, while youth and women had lower social capital levels in terms of influence over local decisions and ability to rely on other community members. Incorporating social capital tools into community health needs assessments in low-income settings provides a valuable overview of community dynamics before project implementation, and Monitoring & Evaluation indicators which allow changes in social capital to be measured at different stages of the project.

## Introduction

Healthy Settings, a key component of Malawi’s Health Sector Strategic Plan (HSSP) 2011–2016 [1], is the World Health Organization’s (WHO) holistic community-led approach to achieving health improvement by addressing social determinants of health, an approach which is central to the current WHO framework on integrated people-centred health services [2]. Healthy Settings projects by their construct have many different components which vary from one group and community to another depending on their priorities: from housing, hospital improvements and waste management to “softer” interventions like leadership skills training and health promotion. It can be challenging to find relevant indicators to monitor and assess the impact of such a complex holistic project, this paper explores if social capital data can provide useful impact assessment indicators at the start of such a project.

Since the 1990’s, social capital has gained recognition in global public health and health promotion theory and practice, as a useful measurement to include in health surveys, project design, and monitoring and evaluation (M&E) tools due to its positive impact on, and connections to, health, wellbeing and social determinants of health [3]. While definitions of the concept vary widely [4,5], in public health literature the term social capital combines together many elements of what makes humans interact with one another, both positively and negatively, and usually encompasses community bonding, social support/networks, trust, reciprocity and community and civic engagement. Grootaert [6] referred to social capital as “the glue that holds societies together”. With its roots in 19^th^ century sociology, the concept of social capital was popularised in the 1990s by American political scientist Robert Putnam who viewed social capital as a “producer” of civic engagement [7], and subsequently social capital theory has been adopted by many different disciplines including public health, public policy, international development and economics. [8].

The multi-disciplinary interest in social capital as a concept and measurement tool has led to a wide variety of theoretical approaches to assessing social capital within public health research over the past 20 years [4]. Of particular interest, with respect to the Healthy Settings project in Malawi, is the discourse about potential links between strong social capital, coping strategies and community participation [9, 10]. It has been documented that if poor communities have high levels of social capital, for example they can rely on their neighbours in times of illness, they trust that local leaders will represent their interests, or they can pay later at their local shop for medications they need, that they are less vulnerable and are more likely to have coping strategies that address the daily challenges that poverty brings, and are more likely to participate in community development [6].

Measuring social capital as part of monitoring and evaluation of community health projects includes understanding and putting a value on Grootaert’s “glue”, and on complicated notions like our sense of home, or who we can rely on in a disaster [6]. A better understanding of social capital through measurement and data collection could help to address wide-ranging social and economic challenges in low-income settings [9,11].

The social capital data examined in this paper was collected in Mfera, Chikwawa district, Malawi as part of a Healthy Settings participatory needs assessment carried out by Scotland Chikwawa Health Initiative (SCHI) in 2014. Consistently listed as one of the least developed countries in the world [12], Malawi has attracted large numbers of non-governmental organisations (NGOs) and other development organisations over the years, many working on health initiatives that aim to improve the health and wellbeing of communities. Yet communities in this country continue to face a wide range of challenges in terms of social determinants of health: from food insecurity, low accessibility to safe water and sanitation, high morbidity and mortality rates to low income levels, unemployment and rising crime [13], all of which are documented by local and national government, academics, NGOs, donors and the international community. SCHI’s Healthy Settings community health project aims to address a wide range of these challenges and is therefore a useful case study for examining whether a better understanding of social capital can have a beneficial impact on tackling the social determinants of health in low-income settings like Malawi.

To date, there are no peer-reviewed reports of social capital data collection within health project needs assessments in Malawi. However, a systematic review of social capital literature, including 22 health related studies, highlighted the current popularity of social capital in public policy in South Africa [14]. In reviewing 15 social capital measurement tools, mainly developed in the USA and UK, the review reported a lack of academic literature on measurement tools developed in Africa for low-income communities. The review also echoes other social capital research that there is an on-going need to trial more innovative methods to understand and measure social capital effectively in low-income settings [15,16]. In response to these gaps, the current study adapted one element of the Schutte tool, the Community Index (C-Index), for collection of social capital data as part of The Scotland Chikwawa Health Initiative’s (SCHI) extensive needs assessment, prior to the implementation of the 3-year Healthy Settings project in rural Malawi.

The needs assessment aimed to produce profiles for each community involved in the project from data collected using mapping, transect surveys, household surveys, and Focus Group Discussions (FGDs). SCHI chose the Schutte tool to collect the majority of the needs assessment data because the tool had been developed in South Africa by Professor de Wet Schutte in 1993 for use in low-income communities, to measure community priorities and bonding and encourage participation in community health projects [17]. The wider reasons for selecting the Schutte tool for the Healthy Settings needs assessment were based on the participatory focus of the tool, with its theoretical basis in community development theory. The Schutte tool methodology encourages community ownership of projects and has potential to address lack of long-term sustainability identified in community health project evaluations in Malawi, data in S1 Text. By involving the community in the development process, from the needs assessment stage, the aim was to understand community priorities and social capital levels at that point in time, and potentially begin to improve bonding and social capital levels by involving the community in the needs assessment.

Using data gathered during the Healthy Settings participatory needs assessment, this paper will explore social capital levels in 18 project communities amongst specific age, status and gender groups, as well as the community as a “whole”, and discuss the initial implications of these findings on project implementation. The paper will also explore the advantages and disadvantages of the Schutte tool for measuring social capital in the Mfera setting and outline future research into its validity as a social capital measurement tool for low-income settings. By developing new methods to assess social capital, it is hoped that the data in this study will enable researchers to add clarity, as well as a new local setting, to debates about social capital’s contribution to health and resilience.

## Methodology

The theoretical approach that determined the wider research methodology is Arnstein’s ladder of participation [18]: exploring to what extent community members want to/can participate in community development and levels of power sharing between communities, health workers and project staff. The social capital research shares this approach and takes an epistemological position based on interpretivism, i.e. understanding the world from the point of view of study participants [19].

For this study, the Community Index (C-Index) element of the Schutte tool was adapted to measure social capital because three social capital indicators were already embedded—belonging, friendship and reliance. The C-Index in its original form measures community bonding and satisfaction with social determinants of health. In consultation with SCHI fieldworkers and academics from the Department of Environmental Health, University of Malawi—The Polytechnic, small modifications were made to the C-Index to provide researchers with an insight into Mfera’s social capital levels.

Central to the Schutte tool methodology, the Schutte Scale is a hand-held sliding wooden instrument, which for the social capital data collection in this study measured the responses to the predetermined C-Index questions on sense of belonging, friendship, social support, ability to work together and influence. The modified version of the C-Index included three community bonding questions from Schutte’s original C-Index, and a further two social capital questions. The modification of the C-Index also involved translating the tool into the local language Chichewa, ensuring all 5 social capital questions were relevant to the context and cultures in the area under investigation through pre-testing. For the purposes of this study ‘community bonding’ is referred to under the umbrella term ‘social capital’.

### Study design and setting

The study area was in Mfera Health Facility catchment in the north-eastern area of Chikwawa District in Southern Malawi, with a population of 7504 people and 1821 households spread across 18 communities [20].

### Sampling and data collection

Each of the 18 communities was split into six groups (leaders, elderly or marginalised individuals, men, women, male and female youths age 16–24) of 10 members selected at random from their demographic group to ensure they were as representative of the community as possible, and with the aim of hearing traditionally excluded voices (particularly male youth and the marginalised, disabled and elderly). Each member gave informed consent at least one day before the FGD took place and were advised of the location and time to attend. The only exclusion criterion was that participants should not be related to the leadership other than within the specific leaders group. In the youth group, FGD participants with and without children were sought.

Data was collected by four research teams, each made up of an experienced facilitator and a scribe/enumerator, who spent a total of 2 days in each community.

### The Schutte tool: Data collection process

During the FGD process, the Schutte Scale was used to collect quantitative data on the 5 social capital indicators in the modified C-Index. The Schutte scale, a hand-held instrument, was designed to be used by literate and illiterate participants alike during FGDs to provide a quantitative measurement for the C-Index. One side of the instrument is numbered from 1–11 and faces the facilitator, the other side faces the participants and is shaded with light to dark dots corresponding with numbers 1–11 on the other side.

The facilitator asked 5 social capital questions, one by one to measure sense of belonging, friendship, social support, ability to work together and influence, an enumerator recorded responses. The new questions are highlighted in bold:

1. To what extent do you consider this community to be your “home”?
2. How close do you feel to your friends in the community?
3. To what extent can you rely on the rest of the community to come to your aid should you have any problems?
4. **How willing are you to work together?**
5. **How much control or influence do you have over local decisions?**

In response to each question, the participant slid the pointer on the scale to the area matching their satisfaction levels about that question; 11 (dark circles) indicated the highest satisfaction level, while 1 (light circles) was the lowest. The enumerator then recorded the numerical value for each person’s response.

In two communities, Jimuloja and Chimoto, additional qualitative data was collected during the Schutte process on the limitations to working together by probing further about what prevents groups working together, the findings are also documented in the results. The aim here was to determine if social capital qualitative data could provide an additional layer of information and insight on top of the quantitative data; this would be helpful for planning activities at implementation stage and could add to the validity of the social capital tool.

### Data analysis

Data from the 6 groups in each of the 18 communities was analysed using Microsoft Office Excel 2011. The mean response for each social capital question was calculated for each group, and then a group average was calculated for social capital using the group averages for each question. Using the mean figures from each group a community mean was calculated.

The data was then transposed to radar charts to show community profiles of social capital. As part of the participatory process the data was shared with each group and community providing the opportunity for comment and validation.

### Ethics

Written informed consent was obtained from all FGD participants; where participants were illiterate a guardian or household member witnessed on their behalf. All participants were de-identified through the use of codes. Ethical approval for this research was obtained from the National Committee on Research in the Social Sciences and Humanities (NCRSH) through the Government of Malawi’s National Commission for Science and Technology (NCST) and the University of Strathclyde.

## Results

This study used the Schutte tool to measure the levels of social capital in 18 communities in Mfera via 108 FGDs conducted with 722 participants (leaders n = 143; men n = 94; women n = 121; marginalised/elderly n = 127; male youth n = 116; female youth n = 121); limited qualitative data was collected from 2 of the communities.

**Fig 1:**
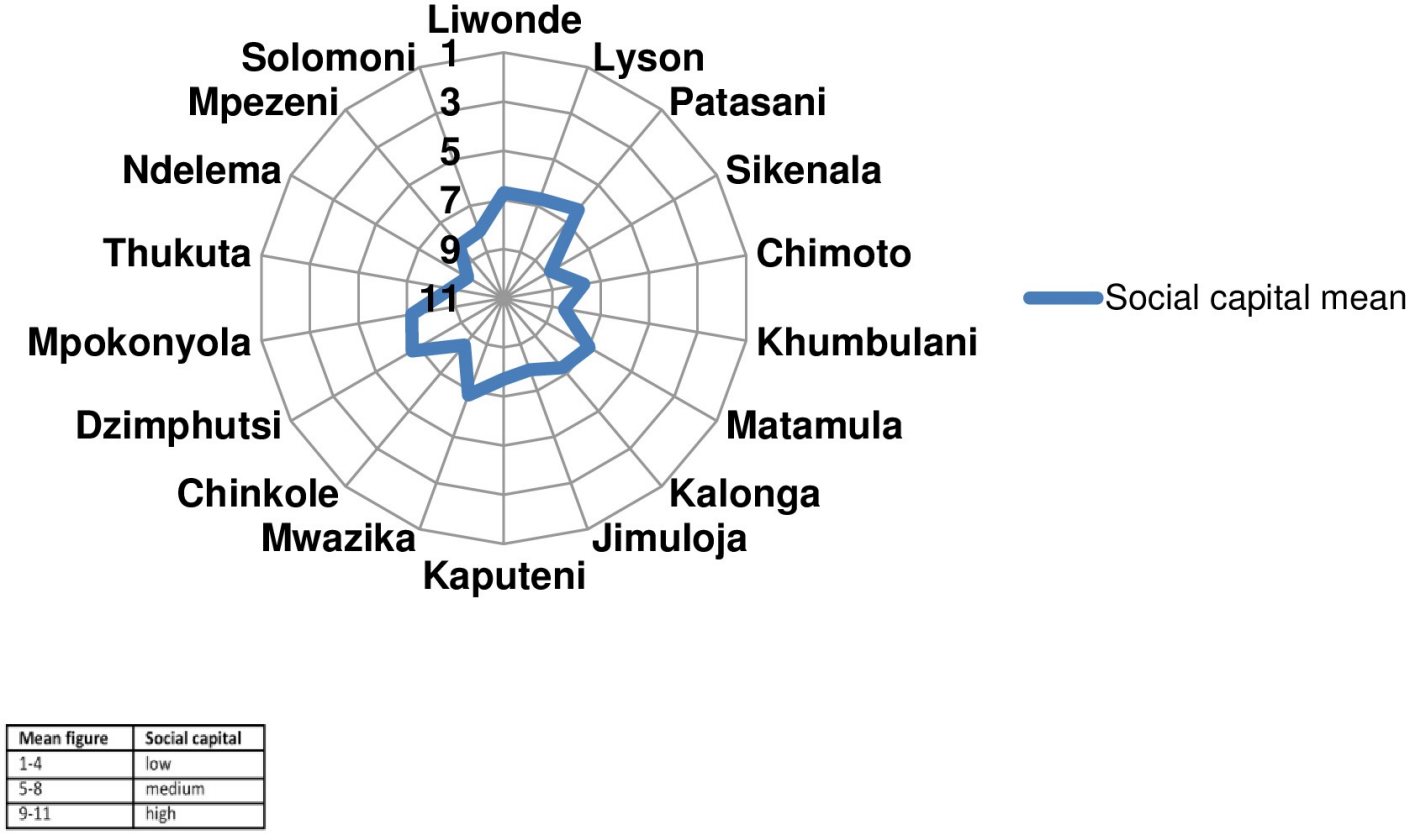
Mean social capital scores for the 18 communities of Mfera involved in the Healthy Settings project.

The mean social capital scores for each community were plotted onto a radar chart, as shown in Fig 1; scoring starts on the circumference, with 1–4 equating to low levels of social capital, 5–8 medium levels, and ends in the centre, with 9–11 indicating high levels of social capital. None of the communities recorded low levels of social capital; most (n = 17) had medium levels, while Ndelema showed high levels of social capital (9.3). The lowest social capital means occurred in Patasani (6.3) and Dzimphutsi, Liwonde and Lyson (6.7) and Mwazika (6.8).

Analysis of the mean scores for each of the five individual social capital indicators across all 18 communities also indicate medium social capital levels, ranging from 8.9 for sense of belonging to 6.6 for reliance. Then looking at each community individually all had a medium to high sense of their community as “home”. However the data are more varied for other indicators particularly for reliance: with very low levels in Matamula (1.7) and Patasani (4.2) ranging to strong medium levels in Solomoni (8.3) and Jimuloja (8.7).

The group mean values collected in the 18 communities also highlight greater variation in social capital levels among the groups than the community’s mean figures allow for. For example, the lowest mean among the different groups across all 18 communities occurred with male youth (6.8) and female youth (7), while leaders (8.4) and elderly (8.3) had the highest mean social capital level of all of the groups. These results demonstrate the variation that may exist when analysing group versus community mean social capital levels.

**Fig 2:**
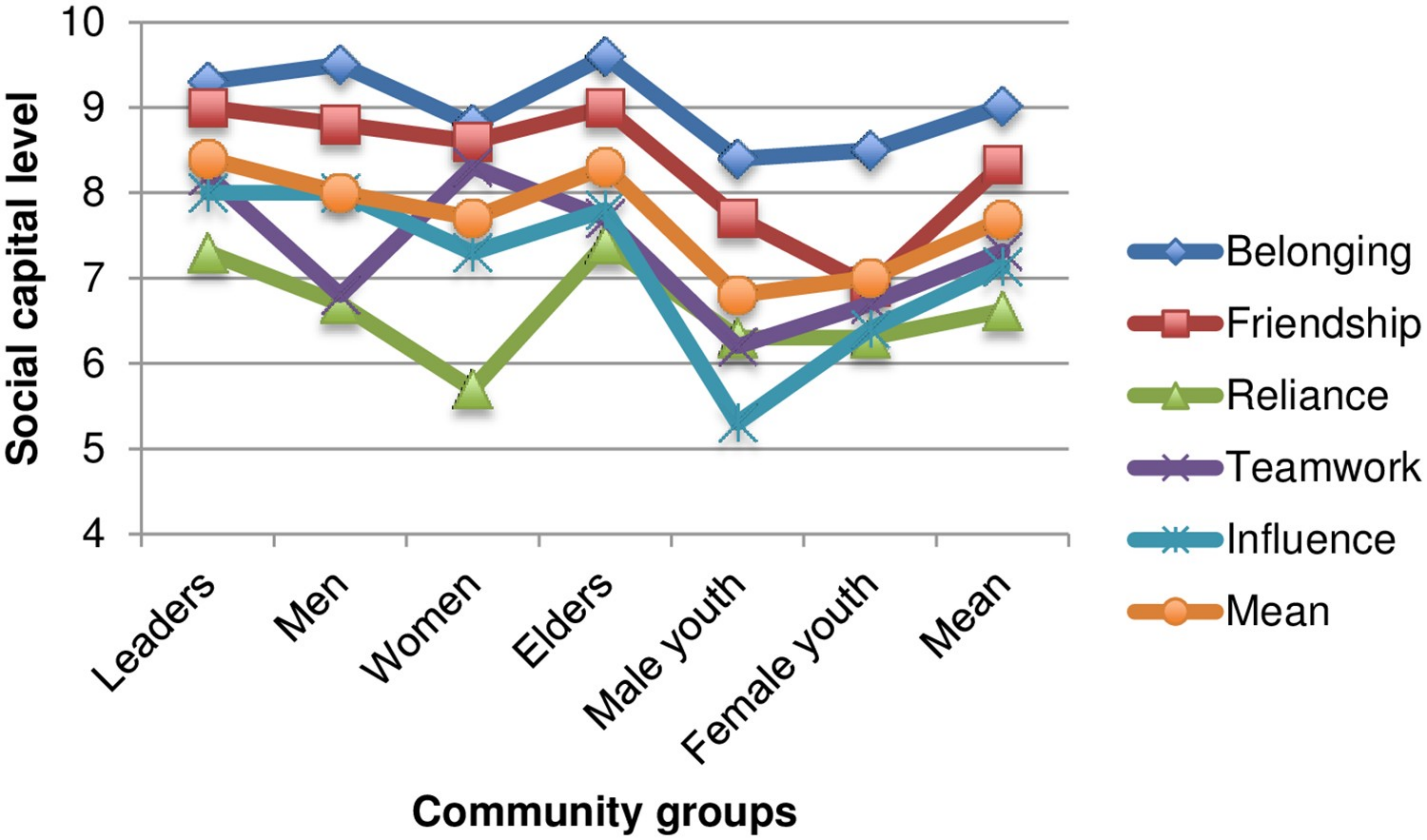
Social capital levels—Community group and indicator.

Examining each indicator by group also highlights variability in social capital, with deficiencies among specific groups again being highlighted. Fig 2 shows that all groups have medium levels of social capital, with high levels for a few groups for belonging and friendship. Both male and female youth have the lowest social capital for each indicator, with the exception of reliance for which women reported the lowest levels of being able to rely on the wider community. For the majority of indicators elders and leaders groups had the highest levels of social capital.

**Fig 3:**
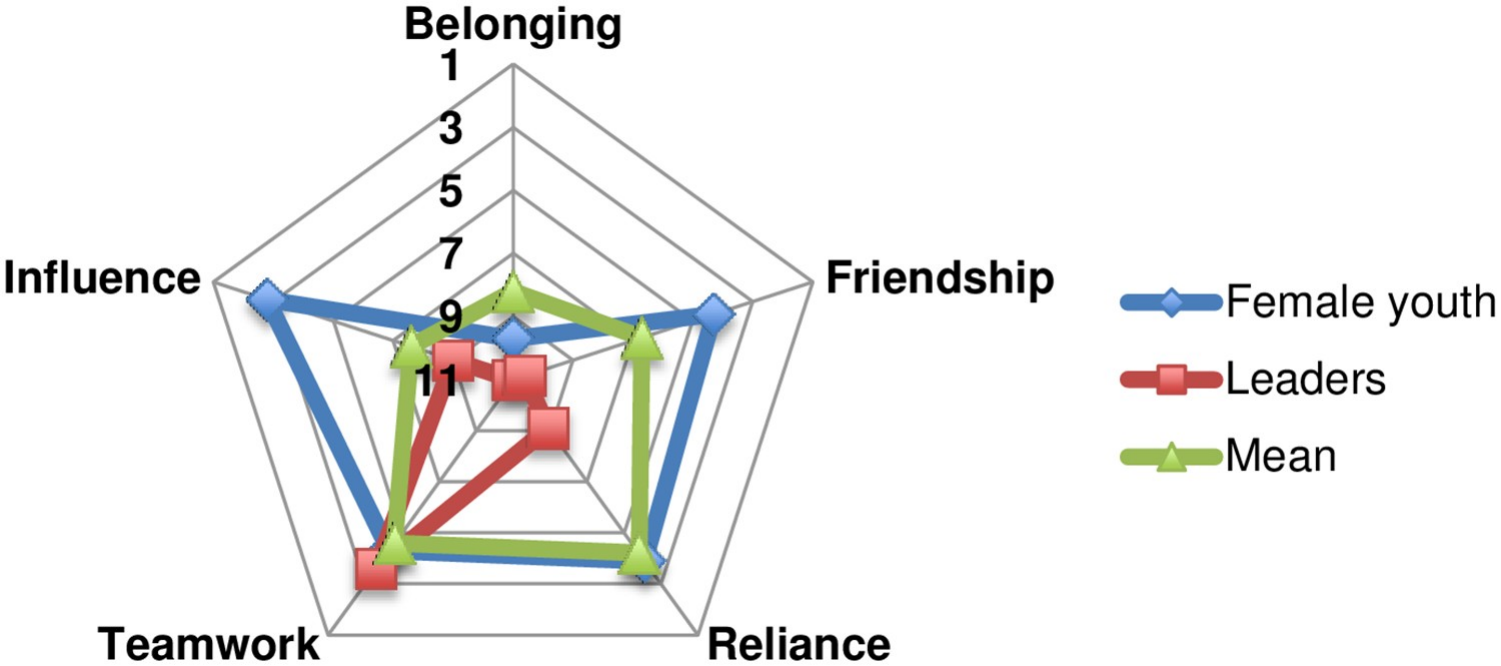
Patasani female youth and leaders: Social capital level indicators.

Further analysis of the data for each community demonstrates the difference in means for the five social capital indicators between groups from the same communities. Taking Patasani as an example (Fig 3), while the sense of belonging was high (9.7 and 11) and ability to work together was weak (4.3 and 3.6) in both female youth and leadership groups respectively, all other indicators varied, for example friendship and reliance were high for leadership (10.6 and 9.1) and low for female youth (4.3 and 3.9). In comparison with the overall mean figures (6.7 and 7.6), female youth are out of synch with all of the other groups in terms of sense of friendship (4.3) and influence (2.8), but like the leaders have a stronger sense of belonging (9.7) than other groups in Patasani.

### Analysis of each indicator

#### Belonging

As can be seen in Fig 2, sense of belonging was the highest social capital indicator amongst all groups, ranging from male youth (8.4) to elders (9.6). From community to community there was more noticeable disparity. The lowest score was registered for Sikenala and Lyson (7.7), and the highest in Thukuta (10.2), Mpokonyola, Ndelema and Mpezeni (9.9). While all groups in Mpokonyola ranked belonging high all neighbouring villages had lower mean scores.

#### Friendship

The mean for friendship was medium (8.3), the second highest social capital mean ranking after belonging. Breaking down by age groups, male and female youth ranked friendship lower than the other groups at 7.7 and 6.9 respectively, and elders and leaders highest (9). The communities who ranked friendship lower were Patasani, Dzimputsi and Liwonde (less than 7.5), while five communities ranked friendship as high: Khumbulani (9.1), Jimuloja (9.7), Thukuta (9.8), Ndelema (9) and Mpezeni (9.5).

#### Reliance

Reliance had the lowest mean ranking of all of the indicators (6.6) with low and medium rankings in all 18 communities. Both female groups and male youth had the lowest satisfaction scores for reliance on the wider community: women (5.7), male youth (6.3), and female youth (6.3), leaders and elders/marginalized groups had the highest scores: 7.3 and 7.4 respectively.

Looking at specific community scores for the reliance indicator 15 communities had medium scores, the communities with the strongest sense of reliance were Jimuloja (8.7), Ndelema (8.5) the largest community in geographical size and relatively isolated compared to others, and Solomoni (8.3) the smallest. Three communities had low rankings: Matamula (1.7), Patasani (4.2) and Liwonde (4.9), in Matamula each group gave a low ranking.

**Fig 4:**
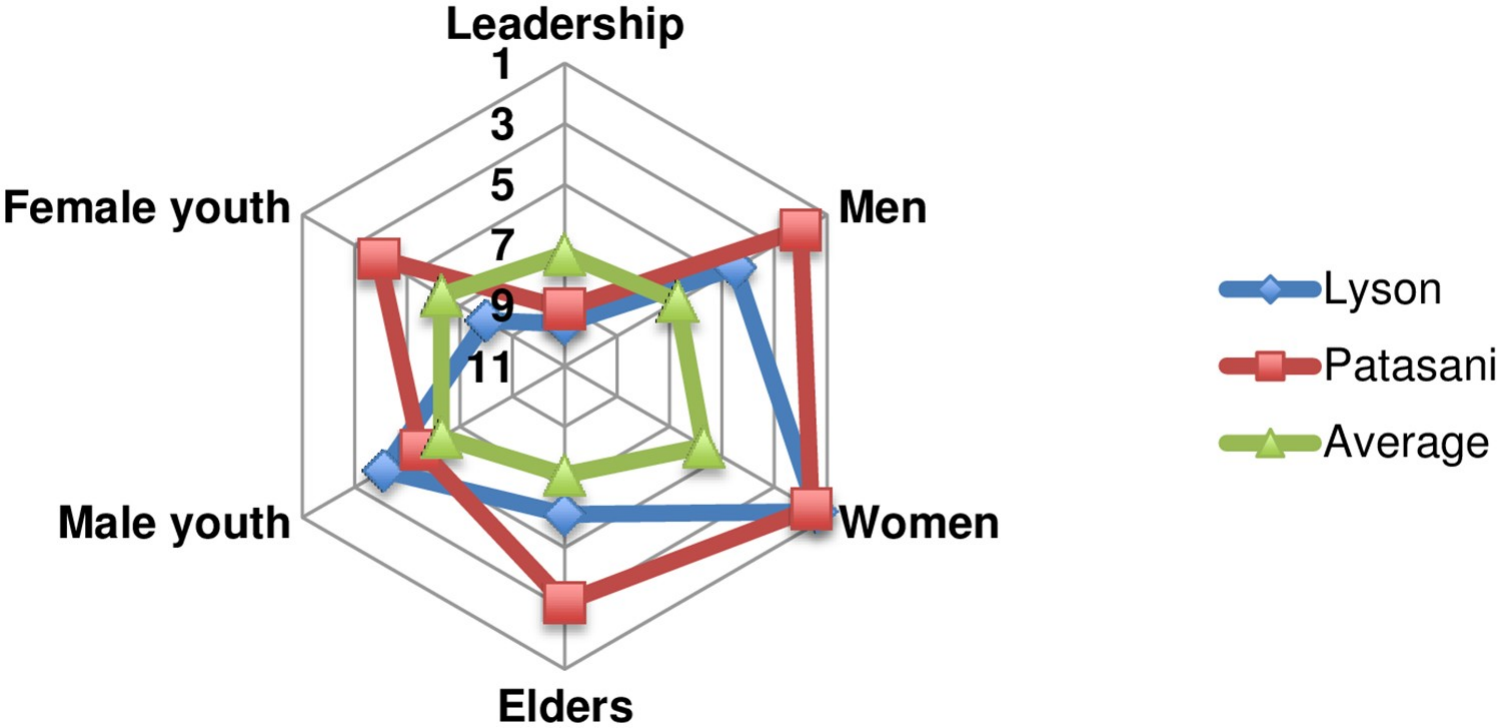
Comparing reliance amongst age/gender and status groups.

It is interesting to note that when looking at the reliance indicator in more detail (Fig 4) that in several communities the disparity between the rankings for reliance on the wider community was important: for example women ranking reliance 1.4 and 1.6 respectively in Lyson and Patasani with leaders ranking 9.6 and 9.1 respectively. The largest disparity was usually but not always with leadership groups, for example in Sikenala reliance was ranked 10.4 by male youth but only 3.8 by men.

#### Influence

The sense that group voices are heard and that they have influence had a medium mean score (7.2) with the lowest amongst the two youth groups: female youth with a score of 6.4 and male youth at 5.3. Mwazika and Jimuloja had the lowest means (5 and 5.4) with particularly low rankings (1.3–4.9) from youth and women’s groups, while the elders and men’s groups in both of those communities scored over 7. As communities Sikenala and Ndelema had high levels of influence (10.4 and 9.2 respectively).

#### Teamwork

All age groups had medium mean scores for teamwork, the groups least willing to work as a team were male youth (6.2) followed by female youth (6.7). A few communities had noticeably low willingness to work as teams: Patasani (4.6) and Mwazika (4.3) and Liwonde (5.4), Lyson (5.3) and Mpokonyola (5.6) had lower medium scores.

Probing questions on limitations to working together, asked in Jimuloja and Chimoto, provided additional insight including a number of the groups referring to some members feeling “superior” to others, disagreements on “what to do”, misunderstandings, poor leadership, hunger and lack of time. Leadership bias towards their own friends and family during food donations was raised as an issue by women, men and leadership groups and affected all aspects of social capital.

During the feedback meetings with each community and group, they concurred with the levels of social capital indicated by their peers and recorded on the radar graphs and community profile, with no changes requested.

## Discussion

According to theorists, social capital can protect poor communities from some of the realities of poverty [9], and like other forms of capital, can contribute to sustainable implementation of community health projects [21]. The use of the adapted Schutte methodology enabled mapping of social capital levels in each of the 18 communities assessed, an activity that has not taken place previously in this population. The mean scores gave an overview of the communities possessing medium to high levels of social capital, before project implementation. The mean data appears to contradict the widely held perception in the NGO community in Malawi that some previous community development projects in the area have failed due to lack of community cohesion/spirit and jealousy. With only limited anecdotal evidence in the Malawian press [22] and no peer-reviewed literature found to date to back this up, further investigation is needed.

Beyond the overall mean data, the results pinpointed communities and community age/gender/status groups where specific social capital indicators ranged from low to high, highlighting that social capital can vary not only between communities, but also within communities depending on age, status or gender. The results also emphasise the complexities of the term ‘community’, especially the dangers of defining communities by geographical boundaries alone and viewing them as a simple, homogenous unit. The Mfera data concurs with other social capital research that factors like gender, age and ethnicity contribute to varied individual perceptions of health and community relationships [9,10]. The data also highlights the dangers of only consulting with the leaders and/or elders in a community during a needs assessment due to time, protocol and budget restraints.

Analysing each community individually, all had a medium to high sense of their community as “home”, which may relate to the stability of the population with minimal transience occurring in the area. However the data is more varied for other indicators particularly for reliance: with low levels in Patasani (4.2) ranging to strong medium levels in Jimuloja (8.7) and Ndelema (8.5). Examining each of the indicators separately can give fieldworkers and communities a greater sense of where social capital “strengths” and “weaknesses” lie in the different communities and groups.

Detailed social capital data analysis can provide a unique insight for all stakeholders before implementation of interventions into often varied and complex community dynamics, which should then influence the development of a realistic programme of project activities for each community and group. In terms of the current Healthy Settings intervention, analysing each social capital indicator in turn for all 18 communities was advantageous i.e. the strong sense of belonging amongst the majority of groups could be harnessed to encourage cooperation in each community. Meanwhile the lack of ability to rely on the wider community, which ranked the lowest of all indicators, as well as lack of influence in decision-making, had to be acknowledged and tackled through relevant team building activities.

Social capital data can give fieldworkers an additional data set to compare to the long list of needs and priorities, as well as insight into how to work with each group. The social capital levels found through the current study informed SCHI’s programme focus and gave insight into varied capacity building needs, and the type of activities to include in the intervention. For example, lack of willingness to work together as a team and to have a voice in the community amongst male youth were incentives behind sports development interventions including the Moyo Wanga, Chisanko Changa (My Life, My Choice) events [23]; development of Village Action Groups with inclusive membership aimed to give a voice to all demographic groups in community development plans; and leadership training and mentorship of government and traditional leadership aimed to support mechanisms of improved communication and planning.

While the quantitative data gave a certain level of useful insight for project implementation, by highlighting which age/gender groups and communities had lower social capital scores, without qualitative data fieldworkers could only use their previous work experience with the communities to account for why this was the case. Although limited, the qualitative data collected from Jimuloja and Chimoto about the challenges of working together, enabled the SCHI teams to have a more detailed insight into how concerns about leadership bias affected women, men and leaders ability to work together.

The community, group and indicator-specific analyses from the Schutte tool before a project begins can provide a starting point for social capital indicators to be used in the evaluation of holistic community health interventions, enabling shifts in levels of social capital to be tracked from the beginning through mid-term to the end of the intervention.

It was key to this social capital research that the data collection tool utilised was theoretically sound, low cost and easy to use in the field by relatively unskilled community health workers who maybe subjected to constant task shifting [24]; the Schutte tool fitted these needs. Schutte credited the simplicity of the tool, the ability to reveal actual need and the reliability regardless of literacy levels, for the tool’s efficiency in collecting data of this nature [17]. Despite limited peer-reviewed literature about the use of this tool in Africa, it has already been used in a variety of circumstances and communities in Southern Africa to collect qualitative and quantitative data. For example it has been widely used by the Western Cape Provincial Government in South Africa to understand community priorities and bonding, as part of project design and M&E of community development and health projects.

As part of a participatory needs assessment, one way social capital data can be validated is through presenting the findings back to each community and group for feedback; in this instance the process enabled SCHI to verify that communities agreed with the social capital and other needs assessment results [25].

Some of the practical challenges of using the Schutte tool were highlighted by the FGD facilitators:

- The time intensive nature of explaining the Schutte tool, while it is described as a simple tool, in many groups the experienced facilitators said it “took time” to explain/and carry out, which resulted in the need to rush FGD sessions.
- Elderly and illiterate participants struggled to understand the Schutte scale, which required more time to explain and use the tool. The efficacy of the tool for this group warrants further investigation because the scale readings were often higher for this group than others.
- There were technical issues with the scale, e.g. elastic bands, which provide the tension at each end of the scale often broke.

In spite of these issues facilitators concluded the Schutte tool was worth the time investment as a source of needs assessment data.

The simple adaptation of the Schutte tool to include only 5 social capital indicators has drawbacks when compared with other tools because it ignores some important indicators that were revealed during project implementation e.g. lack of trust and jealousy amongst community members and levels of motivation among the leadership. In line with previous research this suggests that social capital indicators vary depending on project location, and as such inclusion of a social capital measurement tool within community health projects requires some tailoring for local and cultural context [10,15,16]. However these additions must be fully justified to ensure the tool does not become too unwieldy or complex for health workers.

A further concern is the simplicity of the current version of the tool may not reflect the complexity of social capital. A recent case study from Kenya investigated the different indicators and forms of social capital in vulnerable communities and outlined the importance of going beyond the quantitative data collected in a baseline survey [10]. The paper proposes a conceptual framework measuring bonding, bridging and linking social capitals using mixed methods including qualitative research and social network analysis. In order to validate the Schutte tool, the next stage of the Mfera research will compare the Schutte tool with some of these established methodologies. However careful consideration is required to balance the theoretical requirements with the need to ensure it continues to be a realistic and functional tool for community health workers in low-income settings.

Although data was collected in a group setting and the type of social capital being measured is defined as collective social capital i.e. characteristics belonging to the community or group as a whole [26], the responses are individual. Social capital commentators have highlighted that this aggregation of responses can be “problematic” because social capital is more than just a “sum of individual social capital” [27]. Likewise the results in Fig 1 could be seen to call into question how useful mean data is for a complex social measurement like social capital and the dangers of simplifying such data into a homogenous community view. The participatory element of the tool aims to provide a check on this by incorporating feedback from all participants on the accuracy and relevance of the social capital data and any outliers are recorded.

## Conclusion

The Schutte tool provided an overview of levels of social capital in each community as well as a wealth of more detailed social capital data for each group. The data highlights the importance of analysing data from different age, gender and status groups in each community to understand the complexities of the social dynamics of each community and each group before starting a community health intervention. Using social capital indicators like reliance and social cohesion, which are not intervention specific, is useful for measuring the impact of a holistic community health project by providing additional health insights that complement more traditional health indicators like mortality and morbidity.

The results highlight the importance of avoiding generalisations and negative assumptions about the rural poor and how collecting social capital data during a holistic needs assessment, before the start of a community health intervention, can uncover complexities that need to be addressed if community participation and sustainable community development are going to occur.

Future adaptations of the Schutte tool for social capital data collection will look at ways to reflect a more detailed analytical framework and methodology, incorporating additional locally-relevant indicators, measuring bonding, bridging and linking elements of social capital, including trust and cohesion. The next stage of research will also further validate its use for social capital measurement through comparison with established qualitative and quantitative methodologies. The challenge will be to ensure rigour while maintaining the simplicity of the tool.

The research recommends incorporating social capital tools into Healthy Settings and other community health needs assessments to understand social capital levels at the project outset, to aid implementation and M&E. However, further research is needed to validate the Schutte tool’s potential contribution to social capital measurement in low-income settings.

## Supporting information

S1 Text Draft review report of the National Open Defecation Free (ODF) and Handwashing with Soap (HWWS) strategies. (PDF)
