# Supplementary material for: Social capital insights from Healthy Settings needs assessment in Malawi
Source: PLoS One. 2018 Oct 19;13(10):e0206156. doi: 10.1371/journal.pone.0206156 (PMC6195280; doi:10.1371/journal.pone.0206156)
Supplement: S1 Text — (PDF) [file pone.0206156.s001.pdf]

Draft Review Report of the National Open Defecation Free (ODF) and Hand Washing with Soap (HWWS) Strategies

Submitted to

The National ODF Taskforce

Ministry of Health Headquarters,

Capital Hill

P.O. Box 30377

Lilongwe 3

Submitted by

Dr Steven Taulo (Team Leader)

Centre for Water, Sanitation, Hygiene and Appropriate Technology Development (WASHTED)

University of Malawi,

The Polytechnic, P/B 303, Chichiri

Blantyre 3

Phone +265884588934, Email: staulo@poly.ac.mw

Authors: Steven Taulo, Christabel Kambala, Save Kumwenda, Tracy Morse

January, 2018

Table of contents

Table of contents i

Summary iv

Introduction iv

Objectives iv

Methods iv

Results v

Cross cutting recommendation for future strategy development vii

Abbreviations and Acronyms ix

SECTION 1.0 INTRODUCTION 10

1.1 Background 10

1.2 Summary of achievements and limitations 13

2.0 **Report outline** 17

2.1 Purpose of this report 17

2.2 Specific objectives of the Report 17

3.0 Methodology 18

3.1 Overview of Methodology 18

3.2 Data collection process and tools 20

3.2.1. Stakeholders mapping and analysis 20

3.2.2 Desk Review 20

3.2.3 Field Research. 21

3.3 Consolidation of data 24

4.0 FINDINGS 25

4.1 Effectiveness of the scope, mechanisms and actions applied in the implementation of the ODF/HWWS Strategies 25

4.1.1 Scope, mechanism and action in current strategies 25

4.1.2 Key findings 26

4.1.3 Key recommendations 27

4.2.1 Open Defaecation Free Malawi Strategy 2011 – 2015 28

4.2.2 Hand Washing With Soap Campaign (Strategy) 2011 – 2012 39

4.3 The extent to which different programs, approaches and other cross cutting issues (by Govt, NGOs and developing partners) have contributed to the implementation of the ODF and HWWS strategies 45

4.3.1 Government Frameworks 45
4.3.1 Supporting programmes 46
4.3.3 Model Districts 48
4.4 Gaps in the ODF Malawi Strategy (2011 - 2015) and National Hand Washing with soap Campaign (2011 – 2012) 50
4.4.1 ODF Strategy 50
4.4.1 HWWS Campaign Strategy 53
4.5 The extent that the changing assumptions and indicators impact achievement of ODF Malawi and hand washing behaviours 56
4.5.1 ODF and HWWS Changing Assumptions 56
4.5.2 Cross cutting issues 59
4.5.2 General recommendations from stakeholders for inclusion to updated Strategy 59
4.6 How the strategies link in with other WASH related strategies and approaches 61
4.6.2 Cross Cutting Programmes 61
5.0 CONCLUSION 63

Acknowledgements

This document was funded by UNICEF through the National Open Defaecation Free Task Force. It was prepared by the consultancy team of Dr Steven Taulo, Dr Christabel Kambala, Mr Save Kumwenda and Dr Tracy Morse. The report represents a summary of findings from numerous stakeholders and report collated between September 2017 and January 2018.

Our gratitude is extended to all those who guided and participated in the preparation of this report from the public, private and community sectors from inception to the completion. Without this wide contribution and content this report would not have been possible.

Summary

Introduction

Diarrhoeal diseases pose significant health risks for the rural population and account for 18% of deaths each year in children under the age of 5. Increasing access to basic sanitation and hand washing with soap at critical times are key interventions to the prevention of future diarrhea and cholera cases. The Government of Malawi with its development partners developed the Open Defecation Free (ODF) Malawi (2011 – 2015) and the National Hand Washing Campaign 2011-2012 Strategies, in line with the MDGs to support attainment of its goal to create a clean, safe and healthy environment. As the initial strategies came to an end (2011-2015), the global community entered the new phase of Sustainable Development Goals (SDGs 2016-2030). As such, Malawi also felt the need to up-date the ODF and the National Hand Washing Campaign strategies to be in line with the national sanitation and hygiene targets and SDG Goal 6.2: by 2030. It is against this background that the National Open Defecation Free Task Force (NOTF) commissioned this consultancy whose TORs were to provide a framework for facilitating the review and development of the new National ODF and Hand Washing with Soap (HWWS) strategies. This report is as a result of field data collection, literature review and stakeholder consultation which are informing the revision of the ODF and HWWS strategies to support Malawi in meeting the SDG targets.

Objectives

NOTF outlined specific objectives for this review which have been arranged in two stages as follows:

Stage 1:

- Review of current country strategies with emphasis on original assumptions.
- Review the effectiveness of the scope, mechanisms and actions applied in the implementation of the ODF/HWWS Strategies.
- Review the extent to which different programmes, approaches and other cross cutting issues (by Government, NGOs and development partners) have contributed to the implementation of the ODF/HWWS strategies.

Stage 2

- Examine the extent that the changing assumptions and indicators impact achievement of ODF Malawi and Hand Washing behaviours.
- Examine how the strategies link in with other WASH related strategies and approaches
- Identify gaps in the ODF Malawi Strategy (2015) and National Hand Washing Campaign and incorporate lessons and practical experiences from field application.

Methods

The assignment used a number of data collection methods including stakeholder analysis, desk review, field research and stakeholder consultation detailed as follows:

Stakeholder analysis: Stakeholder analysis was used to identify project's key people with stake, interest or influence in reducing open defecation and promoting hand washing with soap. Stakeholder analysis was a useful tool for identifying people and organizations and institutions that assisted in providing information regarding ODF and HWWS. The information from identified stakeholders was gathered using Key Informant Interviews (KII) and Focus Group Discussions (FGD) during field research and stakeholder meetings.

Desk review: The desk review constituted an important step in the process of reviewing the national ODF and HWWS strategies. It provided the evidence base for the review. Reviewing all documentation (grey, published and peer reviewed information) relating to the issues covered in the existing strategies to develop as complete a picture as possible of the current state of ODF and HWWS both in and outside Malawi. This involved using the following techniques: Internal Desk Research, External Desk Research, Online Desk Research, Government published data and Customer desk research.

Field Research: This involved creation and collection of primary data from the field setting. The process involved determining what precise data was necessary and from where this information needed to be obtained. Field research was performed by the consultancy team in person in 6 Districts and with key stakeholders, through KIIs (n=24) and FGDs (n=38). Purposive sampling was used to recruit participants for both the KIIs and FGDs.

Stakeholder Workshop was undertaken to get provide feedback and validation of the desk review and field results, as well as receive input from further Districts and stakeholders. The workshop used presentations, world café consultation and direct feedback.

Results

Key Findings

Findings demonstrate that there have been positive results and progress towards meeting strategic targets from 2011-2015. Nevertheless there are still significant barriers and challenges to the achievement of key goals of ODF and HWWS across Malawi. The main gaps identified include:

Scope

- The ODF strategy focuses only on the rural population, which has limited the focus and success of ODF achievement.
- The ODF strategy referred only to households with no requirement for ODF status in public spaces and institutions.
- There is no reference or integration of ODF strategy with menstrual hygiene management.
- Neither strategy has specific reference or support for vulnerable and marginalized groups.

Mechanism

- Both the ODF and HWWS strategies do not provide specific definitions of latrines, hand washing facilities, etc. which leads to variation in implementation.
- The ODF strategy does not consider the whole sanitation chain (capture to disposal).

- ODF strategy implementation was to be overseen by the NOTF which represents the Ministry of Health and the Ministry of Agriculture, Irrigation and Water Development with key development partners and civil society which doesn't include other ministries.
- The current ODF strategy is limited to the use of Community Led Total Sanitation (CLTS) and sanitation marketing and does not take into consideration the use of other participatory approaches such as Participatory Hygiene and Sanitation Transformation (PHAST) and mechanisms to achieve ODF.
- Although there is the inclusion of 2 levels of ODF status in the strategy (i.e. 1- appraising a community towards the attainment of the ODF status; 2- sustenance of ODF status after attaining the ODF status), there is little reference to the effective use of the sanitation ladder to achieve continued improvement and sustainability.
- HWWS strategy uses health facilities and schools as key locations for good practice and development of agents of change, but in many cases these were identified as having the poorest standards.
- The use of Health Surveillance Assistants (HSAs) in the drive for ODF was reported as inconsistent across partners.
- CLTS was seen as a 'project' by HSAs, and once partners were gone the implementation also stopped.
- HSAs were used to receiving allowances to undertake this work and therefore stopped their CLTS/ODF activities when they became routine activities.
- Data was inconsistently reported and in some cases validity is called into question.
- In the implementation of both strategies there has been a focus on infrastructure with little concentration on behavior change communication for sustained change.

Key recommendations

Scope

- The scope of the strategies should include proper definitions of a latrine (including menstrual hygiene management) and hand washing facilities, and should consider the whole sanitation chain.
- Areas must ensure ODF and HWWS in all households and public areas and institutions before they can be declared ODF.
- New strategies must tackle both urban and rural populations.
- Support for vulnerable and marginalized populations must be more effectively integrated.
- Integration of menstrual hygiene management
- Criteria and mechanisms for being declared ODF should be reviewed.

Mechanism

- NOTF should be more multidisciplinary in its membership with the inclusion of representatives from nutrition, disabilities and other appropriate government departments to ensure integration of services.
- Effective sanitation marketing and financing models need to be more fully integrated into CLTS triggering programmes.
- Movement towards a requirement for standard systems to be constructed should be considered which would improve quality of latrines and create business for masons and entrepreneurs.

- Training of masons should be linked to technical training colleges and schools.
- ODF must be incorporated into the routine activities of HSAs without the requirement of allowances.
- Funding must be ring-fenced for ODF activities from the District budget.
- Stakeholders reported the need for integration in community structures for effective implementation, and the valuable role of Natural Leaders. They suggested a continued use of traditional and natural leaders to support the implementation, achievement and sustainability of ODF status. It was also suggested that natural leaders and their roles in community sanitation and hygiene achievement should be recognized.
- Vulnerable and marginalized groups should be engaged from the offset of the CLTS programme and be involved in the training, implementation and verification processes to ensure appropriate systems are in place to support them.
- By laws should continue to be encouraged but must be enforced consistently for all community members and be facilitative rather than punitive, taking into consideration human rights.
- Large ODF celebrations attended by the Minister and dignitaries should only take place when the District has achieved ODF status.
- School WASH standards need to be completed and circulated to ensure improvement at facilities. These standards must include a range of low cost HWF suitable for school settings.
- The concept of using schools and children as agents is still a welcome one but needs better integration and structure
- Health facilities must be supported to ensure that they are modeling improved sanitation and HWWS to promote good behaviour.
- HWWS promotion needs to be integrated into all relevant clinics, e.g. antenatal, growth monitoring, immunisations, OPD, etc.
- Behaviour change messaging needs to be developed based on sound principles and with an understanding of the audience and behavioural factors which are being targeted.
- The need for, and promotion of HWWS requires effective public private partnerships and these require to be engaged on a more regular and formal basis.
- Strengthen CLTS and HWWS monitoring systems: There is need for more detailed monitoring and evaluation of progress and effectiveness.
- Improved integration of behavior change communication to support sustained improvements in ODF and HWWS throughout Malawi.

Cross cutting recommendation for future strategy development

It is clear from the feedback from all stakeholders and desk review, that future strategies must address concerns regarding integration of sanitation and hygiene programmes to ensure sustained change across Malawi and achievement of the SDGs by 2030.

With this in mind, it is the overall recommendation of this review that the current ODF and HWWS strategies should be integrated into a more general 'hygiene and sanitation' strategy. This would support not only the integration of HWWS and ODF programmes, but also the inclusion of key issues raised in stakeholder meetings such as menstrual hygiene

management and solid waste management (including faecal sludge management). This would be an all encompassing strategy which targets rural and urban populations, domestic houses, commercial premises and institutions across the country. Only then can Malawi truly meet the target of Universal Sanitation and Hygiene for All.

Abbreviations and Acronyms

ASHPP	: Accelerated Sanitation and Hygiene Practices Programme
CLTS	: Community Led Total Sanitation
GSF	: Global Sanitation Fund
HWWS	: Hand Washing With Soap
JMP	: Joint Monitoring Programme
MDGs	: Millennium Development Goals
MEHA	: Malawi Environmental Health Association
MoAIWD	: Ministry of Agriculture, Irrigation and Water Development
MoLG	: Ministry of Local Government
MOH	: Ministry of Health
NGO	: Non-Governmental Organization
NOTF	: National Open Defecation Task Force
NSHCU	: National Sanitation and Hygiene Coordinating Unit
NSP	: National Sanitation Policy
OD	: Open Defecation
ODF	: Open Defecation Free
SGDs	: Sustainable Development Goals
SLTS	: School Led Total Sanitation
TOR	: Terms of Reference
UNICEF	: United Nations Children's Fund
WASH	: Water, Sanitation and Hygiene
WASHTED	: Centre for Water, Sanitation, Hygiene and Appropriate Technology
WESNET	: Water and Environmental Sanitation Network
WHO	: World Health Organization
WSSCC	: Water Supply and Sanitation Collaborative Council

SECTION 1.0 INTRODUCTION

1.1 Background

Morbidity and mortality from diarrhoeal disease continues to be a significant burden to the global population. With the main burden falling on children under the age of 5, it has been estimated that 360, 000 deaths from diarrhoea per year can be attributed to environmental contamination and exposure (Figure 1). Infection could be caused through numerous environmental routes as depicted by the exposure pathways diagram (Figure 2) (Bartram and Cairncross, 2010). However it is clear that following enteric pathogen excretion, effective sanitation is integral to the reduction of disease transmission. In 2012, it was estimated that 280,000 people, mostly children under five years old, died from diarrhoea caused by lack of basic sanitation (Prüss-Ustün et al. 2014).

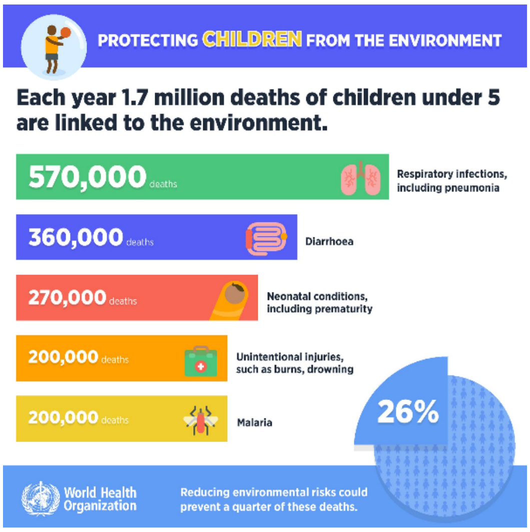

Figure 1: WHO estimates of childhood illness from environmental exposure?

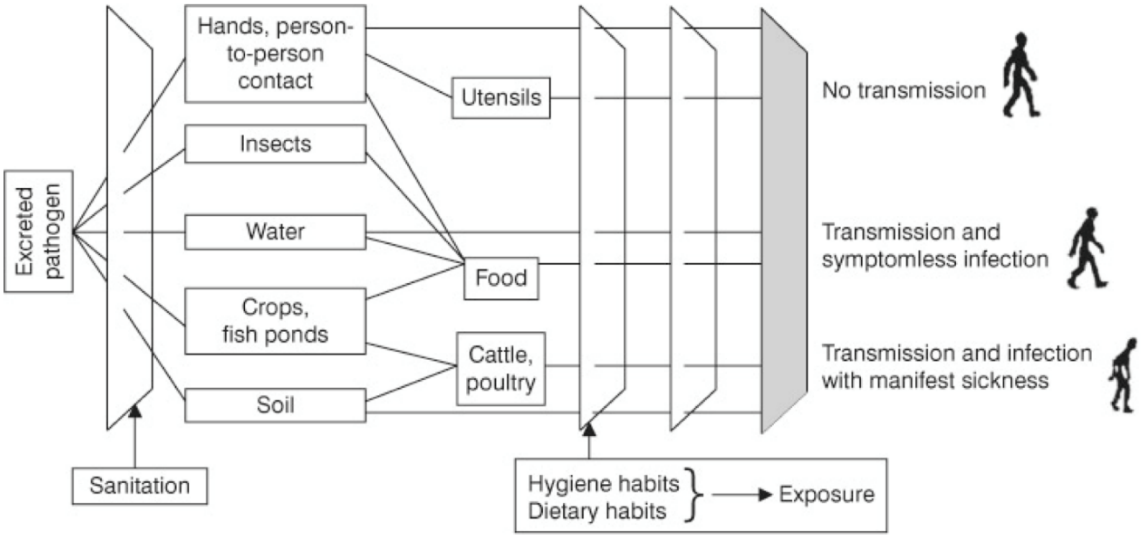

Figure 2: Various transmission routes of pathogens from extra of an infected person to a healthy person (Source: Pond, 2015)

These deaths could be prevented in part, by improving access to safely managed sanitation and improved hygiene practices (Brown et al. 2014).

Hand washing with soap at critical moments, such as before eating and after defaecation, can also prevent infectious diseases by interrupting the transmission of infectious agents. Empirical evidence suggests that handwashing with soap reduces the risk of diarrhea by 47% (Curtis and Cairncross, 2003, Cairncross, 2010), acute lower respiratory infections by up to 34% (Luby et al, 2005), and soil-transmitted helminths (Strunz et al, 2014), and it has been recognized as one of the most cost-effective health interventions to reduce the burden of disease (Bartram and Cairncross, 2010). Yet, only 19% of the global population is estimated to wash their hands with soap after using sanitation facility or handling children's excreta (Freman et al, 2014).

Good hygiene is of vital importance in Malawi due to the lack of basic sanitation in the country. Evidence of actual hand washing practice is scanty but studies in rural areas suggest that the actual practice of HWWS at key times is between 3 – 18% but more likely on the low end of this scale, as responses tend to exaggerate actual and regular practice. Observations in Malawi show that HWWS promotion is undertaken as an ad hoc activity both at national and local level. Current efforts to promote good hygiene and HWWS in particular, have not been sufficient to bring about mass behaviour change on the scale that is needed. Efforts producing piecemeal village-by-village and pilot approaches have had some impact in Malawi but nothing on a large or national scale has been attempted (MoH, 2015)

The Joint Monitoring Programme for Water Supply and Sanitation estimates that 2.3 billion people globally lack access to basic sanitation (use of improved sanitation facilities that are not shared with other households) and that 892 million people practice open defecation (WHO/UNICEF 2017). As such the control of open defaecation is a primary public health concern in the reduction of diarrhoeal disease morbidity and mortality, and the spread of diseases such as cholera across vulnerable populations.

In recognition of this priority, the Government of Malawi launched the National Sanitation Policy in 2008. This policy emphasized the need for **sanitation for all in Malawi.** It envisioned a transformed country where all the people have access to improved sanitation, and where safe hygienic behaviour is the norm. This included the recycling of solid and liquid waste, leading to a better life for all the people of Malawi, through healthier living conditions, a better environment and a new way for sustainable wealth creation (National Sanitation Policy 2008).

To facilitate achievement of this Policy, a number of strategies were developed to support the Government of Malawi to meet its sanitation and hygiene goals under the Millennium Development Goals and Malawi Growth and Development Strategy.

Key to this was the development and launch of the Open Defecation Free Malawi (ODF) 2011-2015 Strategy and the National Hand Washing Campaign (2012). In line with the MDGs they aimed to support attainment of its goal to create a clean, safe and healthy environment, which results in improving people's health and wellbeing. The strategies were developed to ensure complete elimination of open defecation (OD) and promote Hand Washing with Soap (HWWS) to reduce the spread of sanitation and hygiene related diseases such as diarrhoea and cholera.

Recognizing that increasing access to basic sanitation and use of safe hygiene practices is the main key to the prevention of future diarrhoea and cholera cases, both the National Hand Washing Campaign and Open Defecation Free Strategies were implemented through a range of programmes with implementing partners.

The primary programme was the Accelerated Sanitation and Hygiene Practices Programme (ASHPP). This was a government-led national initiative on sanitation and hygiene funded by the Water Supply and Sanitation Collaborative Council (WSSCC) through the Global Sanitation Fund (GSF). It aimed to support communities to adopt and use safe hygiene practices by eliminating open defecation and increasing their access to improved sanitation.

This programme primarily aimed to achieve the objectives of the Open Defaecation Free Strategy (2011 – 2015) through the Government of Malawi and a range of development partners. These activities have been coordinated by the National Sanitation and Hygiene Coordinating Unit (NSHCU) (Government of Malawi) and the National Open Defaecation Free Task Force (NOTF) (Multisectoral).

Box 1: ODF Declaration Status

Level 1

- 95% of the households must have latrines
- All available latrines must offer privacy, good state of repair, with good roof
- All latrines must show evidence of being used
- All households must properly dispose baby's feacal matter
- No sign of open defeacation in the area
- 5% sharing of latrines is allowed

Level 2

- 100% of the households must have latrines
- All latrines must offer privacy, good state of repair, with good roof
- All latrines must show evidence of being used
- All households must properly dispose baby's feacal matter
- No sign of open defeacation in the area
- No sharing of latrines is allowed
- All latrines must have hand washing facilities

As the initial strategies came to an end (2011-2015), the global community entered the new phase of Sustainable Development Goals (SDGs 2016-2030). As such, Malawi also felt the need to up-date the ODF and HWWS strategies for the national sanitation and hygiene targets in line with SDG Goal 6.2: by 2030, achieve access to adequate and equitable sanitation and hygiene for all and end open defecation, paying special attention to the needs of women and girls and those in vulnerable situations. It is against this background that this review report has been prepared.

1.2 Summary of achievements and limitations

According to the most recent WHO/UNICEF Joint Monitoring Programme (JMP) figures (2015), between 1990-2015, the percentage of the country's population practicing open defecation decreased from 29% to 4% and access to improved sanitation increased from

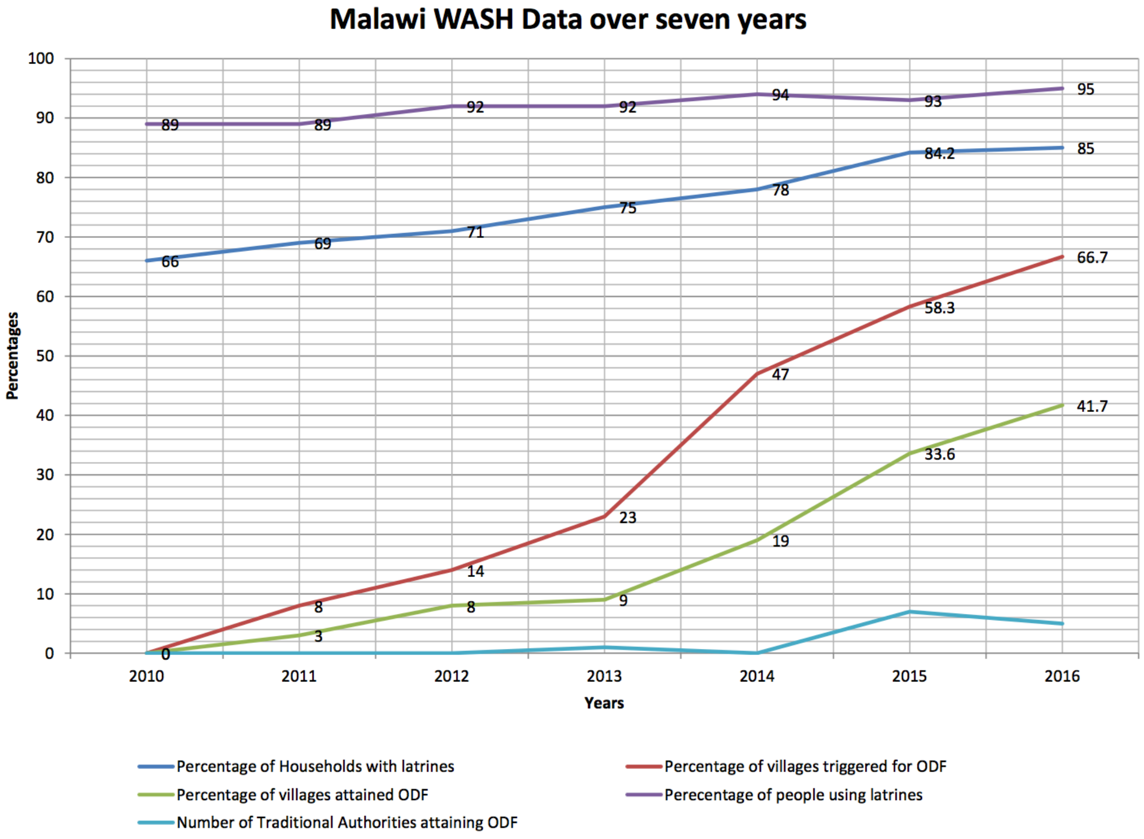

Figure 3: Malawi National WASH Data 2010 - 2017

29% to 41%. Over the period of implementation of the ODF strategy (2011 – 2015), this coverage of latrines has increased from 66 – 85% of households, and use of a latrine has increased from 89 – 95% reported (Figure 3). These national figures, however, mask significant disparities between:

- Urban areas and rural areas, the latter being where the majority of Malawians live, but has not been a focus of the ODF strategy implementation.
- Districts which have achieved significant (100% ODF) and minimal (0% ODF) progress towards targets (Figure 4)
- The achievement of Level 1 ODF status and Level 2 ODF which requires the presence of improved sanitation and effective hand washing with soap facilities and practices (Figure 5)(Box 1).
- Verification as ODF followed by slippage to open defaecation

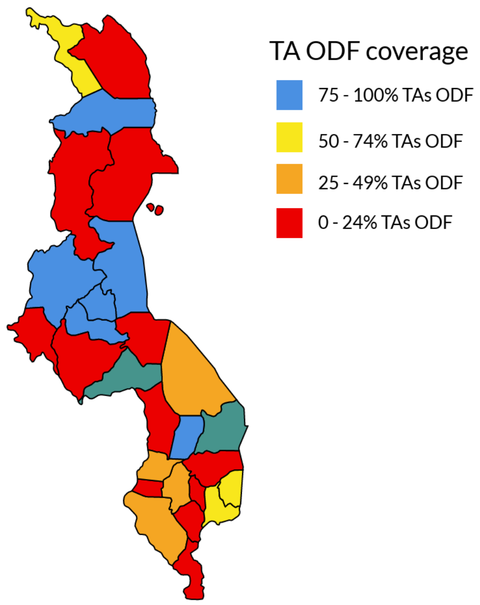

Figure 4: Map of Malawi depicting ODF status by percentage of Traditional Authorities

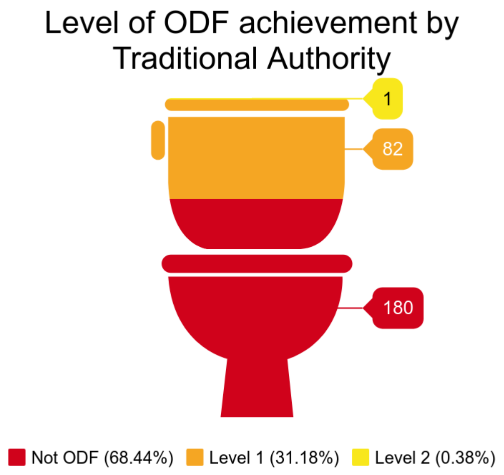

Figure 5: Info graphic depicting the percentage of Traditional Authorities achieving ODF by Level (Box 1)

Achievement of this increased coverage has mainly been achieved through the following mechanisms over the last 20 years:

- Health Education and Sanitation Promotion (HESP)
- Participatory Hygiene and Sanitation Transformation (PHAST)
- Community Led Total Sanitation (CLTS)
- School Led Total Sanitation (SLTS)
- Sanitation marketing

Significant emphasis has been placed on the use of CLTS throughout the ODF strategy implementation, following the standard process steps of:

- Pre triggering preparations
- Triggering of Villages
- Post triggering (follow ups)
- Verification
- Declaration and celebration

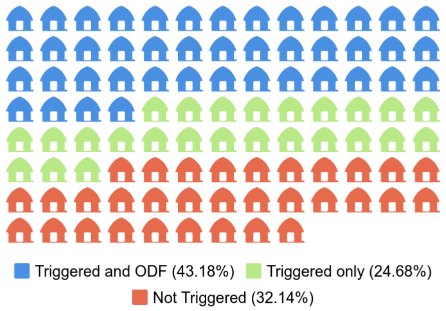

Figure 6: Percentage of village which have been triggered for CLTS and achieved ODF in Malawi (2017)

Malawi has 38, 362 villages of which 68% have been triggered. As a consequence of this triggering 43% of the villages in Malawi have been declared ODF (Figure 6). At Traditional Authority (TA) level, this equates to 57% triggered and overall 32% declared as ODF with only one of these as Level 2 (Figures 4 and 5)

Emphasis to date has primarily been placed on the achievement of ODF status at Level 1. As such this has excluded the achievement of HWWS to a large extent, as it does not require

the existence of hand washing facilities (Figure 7). This is reflected in the low progress in increasing coverage of hand washing facilities across Malawi with a less than 10% increase to 34% achieved by 2016.

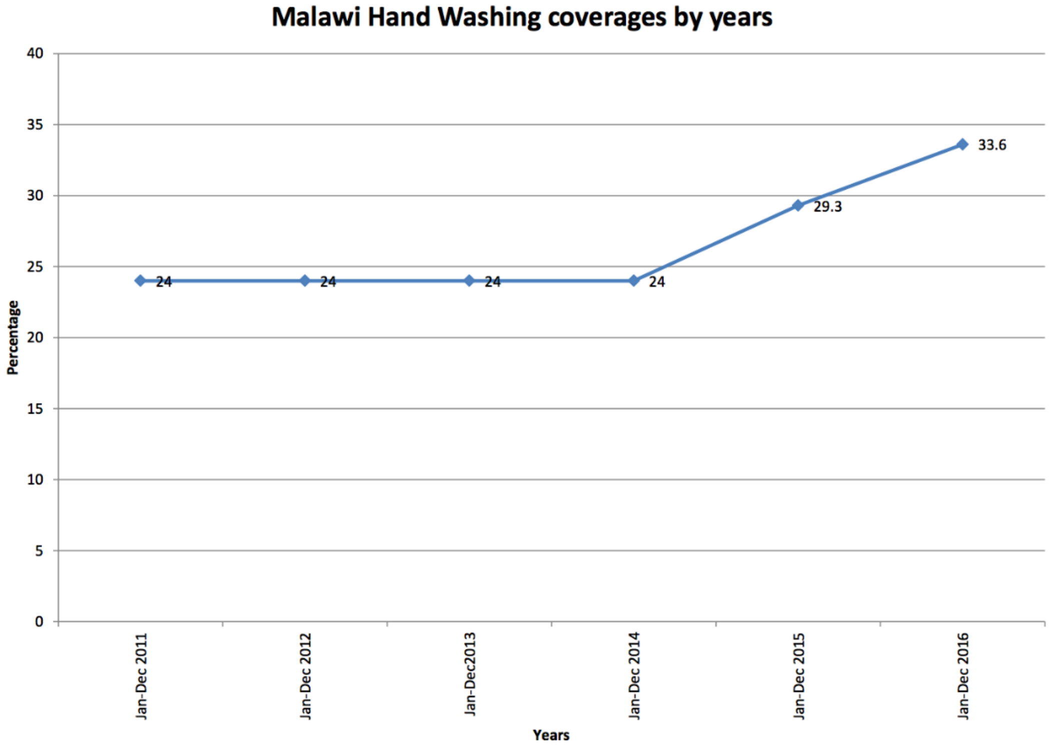

Figure 7: Percentage of latrines with hand washing facilities in Malawi (2010 - 2016)

As with ODF this coverage varies significantly across the country (Figure 8), and statistics are also subject to debate, as the presence of a hand washing unit does not necessarily reflect consistent use at high risk times. The lack of soap available, and the location of the hand washing facility by the latrine can also negate effective HWWS practice at critical times thereby achieving the required reduction in disease transmission. It must also be considered in Figure 8, that many of the Districts showing high coverage of hand washing facilities have not achieved ODF and as such do not have a latrine at >90% of households.

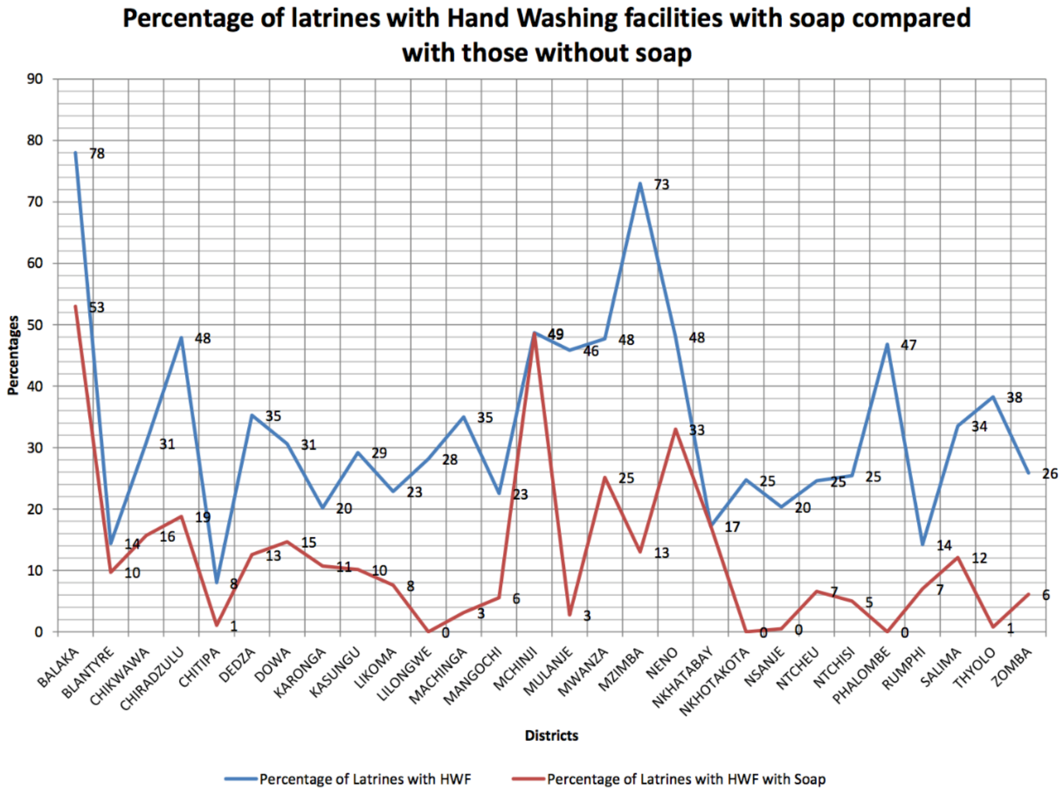

Figure 8: Percentage of latrines with a hand washing facility compared to those with soap

1.3 Conclusion

Although considerable strides have been made since the inception of the ODF and HWWS strategies in Malawi (2011), there is still a significant way to go if the country is to meet SDG 6.2, and ensure safe and effective practices nationwide to reduce diarrhoeal disease.

Primary focus has been on the provision of infrastructure through CLTS and mass media campaigns. Although progress in this area is still needed, it must also be accepted that these activities require to be complemented with appropriate health promotion and behaviour change programmes.

2.0 Report outline

2.1 Purpose of this report

The primary purpose of this report is to support the review of the ODF Malawi (2015) Strategy and National Hand Washing with Soap (HWWS) Campaign Strategy; and to guide the development of new national strategies based on the lessons learned in line with the Sustainable Development Goal (SDG) and Malawi Growth and Development Strategy.

2.2 Specific objectives of the Report

The objectives covered within this report fall into two clear stages:

Stage 1:

- Review of current country strategies with emphasis on original assumptions.
- Review the effectiveness of the scope, mechanisms and actions applied in the implementation of the ODF/HWWS Strategies.
- Review the extent to which different programmes, approaches and other cross cutting issues (by Government, NGOs and development partners) have contributed to the implementation of the ODF/HWWS strategies.

Stage 2

- Examine the extent that the changing assumptions and indicators impact achievement of ODF Malawi and Hand Washing behaviours.
- Examine how the strategies link in with other WASH related strategies and approaches
- Identify gaps in the ODF Malawi Strategy (2015) and National Hand Washing Campaign and incorporate lessons and practical experiences from field application.

These objectives cannot stand alone and as such the linkages are outlined in Figure 9.

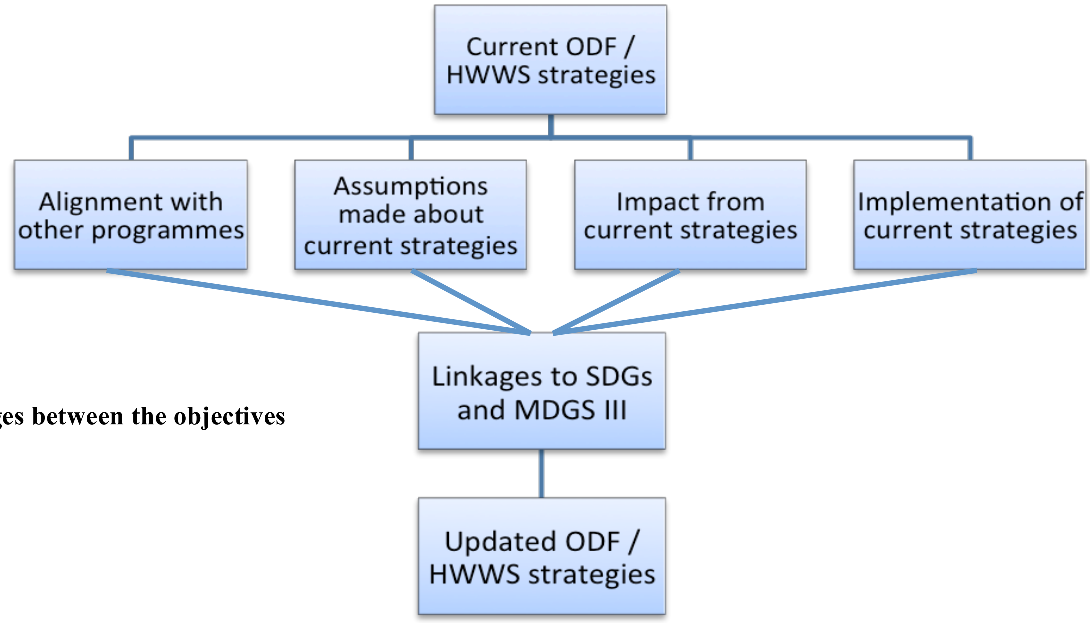

Figure 9: Linkages between the objectives

3.0 Methodology

3.1 Overview of Methodology

The purpose of the outlined methodology is to achieve a critical understanding of the key elements of the ODF and HWWS strategies

It is understood that although ODF and HWWS are inextricably linked, they also require standing alone to ensure their integration and adoption with other cross cutting areas. As such, where possible the report outlines the ODF strategy and HWWS strategies separately although they are linked and integrated where appropriate.

Based on the specific objectives, the methodologies outlined in Figure 11 and Table 1 were utilised.

Table 1: Summary data collection methods

	Objective	Means of getting data
Stage 1	• Review the effectiveness of the scope, mechanisms and actions applied in the implementation of the ODF/HWWS Strategies.	• Desk review<br>• Key Informant Interviews
	• Review of current country strategies with emphasis on original assumptions.	• Desk review<br>• Key informant Interviews
	• Review the extent to which different programs, approaches and other cross cutting issues (by Govt, NGOs and developing partners) have contributed to the implementation of the ODF HWWS strategies	• Desk review<br>• Key Informant Interviews<br>• Stakeholder workshop
Stage 2	• Identify gaps in the ODF Malawi Strategy (2015) and National Hand Washing Campaign and incorporate lessons and practical experiences from field application.	• Desk review<br>• Key Informant interviews<br>• Stakeholders workshop
	• Examine the extent that the changing assumptions and indicators impact achievement of ODF Malawi and hand washing behaviours.	• Desk review<br>• Key Informant Interviews<br>• FGDs<br>• Stakeholder workshop
	• Examine how the strategies link in with other WASH related strategies and approaches<br>•	• Desk reviews<br>• Key Informant interviews<br>• Stakeholder workshop
	• Develop monitoring framework and structures to be in place to ensure proper coordination between national and local authorities	• Key Informant Interviews<br>• Stakeholder workshop

Figure 11: Summary of Methodology

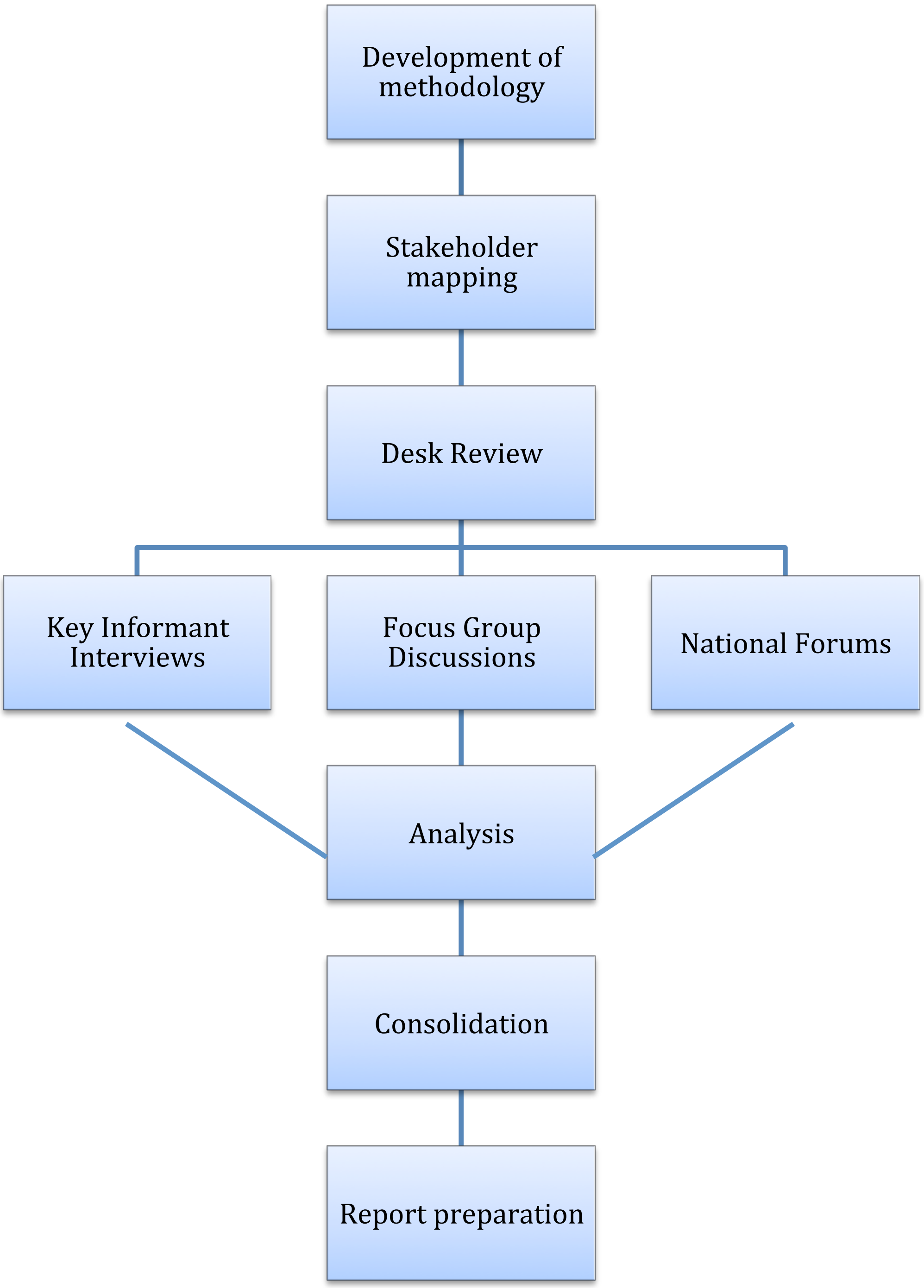

3.2 Data collection process and tools

3.2.1. Stakeholders mapping and analysis

Stakeholder analysis was the identification of organisations with a stake, interest or influence in reducing open defeacation and promoting hand washing with soap. It also involved assessing their interests and the ways in which these interests affected implementation, viability and sustainability. The information from identified stakeholders was gathered through Desk review, Key Informant Interviews and Focus Group Discussions.

3.2.2 Desk Review

The desk review constituted an important step in the process of reviewing the national ODF and HWWS strategies. It provided the evidence base for the review. A review was conducted on all documentation (grey, published and peer reviewed information) relating to current progress in Malawi and where appropriate relate to findings from other countries.

The desk review aimed to document the following:

- The national context of the strategies (key socioeconomic indicators and determinants);
- Progress towards achieving the national targets for impact, outcomes and outputs for ODF and hand washing;
- Investment made and the quality of implementation;
- Factors associated with the performance of the strategies; and
- Information on weaknesses and gaps.

The desk review was conducted using the simple analytical framework presented in Table 2 below.

Table 2: Information required during desk review

Review questions	Information required	Sources of information
Where are we, and are the right things being done?	- Scope (implementation done in a comprehensive manner with attention to inclusiveness, issues, strategies, timelines)<br>- Interventions (CLTS, SLTS, Sanitation Marketing)<br>- Resources	• Administrative sources<br>• Effectiveness studies<br>• Operational research<br>• Resource tracking
Are they being done the right way?	- Assumptions for implementation of the strategy<br>- Delivery models<br>- Participation<br>- Integration<br>- Management<br>- Quality (technical assistance provision to districts)	• Process monitoring<br>• Quality assessment<br>• Operational research
Are the right people	- Coverage (products and services)	• Population based

Review questions	Information required	Sources of information
being reached?	- Behaviour change (ODF status pre and post 2011 strategy)	surveys<br>• Routine reporting<br>• Operational research
Is the programme making a difference?	- Incidence and prevalence of sanitation-related diseases<br>- Latrine and hand washing coverage rates (pre and post 2011 – 2015 ODF strategy)	• Surveillance and surveys

Key sources of information included:

3.2.2.1 National Data and Reviews

The team collated the current national data pertaining to ODF and HWWS. This included the findings and issues raised at the recent National Sanitation and Hygiene Learning Forum (May 2017, Lilongwe) and National ODF Review (November 2017, Lilongwe). These were recorded by the team when they attended the events.

3.2.2.2 Project Reports

Much of the information was generated internally within private organizations, collaborating partners, interested groups and the affected ministries (Health and Agriculture, Irrigation and Water Development). Project documents and NOTF documents (policies, strategies, reports, protocols, regulations and other related documents) were sourced. Where appropriate documents were also sourced from other relevant countries for comparison.

3.2.2.3 Peer Reviewed Literature

A literature review was conducted online to identify the most relevant peer reviewed publications which related specifically to Malawi or had relevant learning to be taken into consideration on the review of the National ODF and HWWS strategies. Where appropriate documents were sourced from other relevant countries for comparison.

3.2.3 Field Research.

This element of data collection allowed the collation of actual and authentic information (primary data) from the field setting. Field research was performed by the consultancy team in person, through Key Informant Interviews and Focus Group Discussions, as outlined below.

3.2.3.1 Key Informant Interviews

KIIs provided explanations and perspectives from the key stakeholders in ODF and HWWS in Malawi. Stakeholders were targeted in 2 key areas: (1) Districts being used for field assessment (n=6) both government staff and development partners, and (2) Central Government offices representing the gatekeepers of the strategy and policy documents (Table 3).

KIIs followed a semi-structured questionnaire (Appendix 1) and were conducted by a member of the research consultancy team.

The questions covered a range of issues:

- ODF and HWWS successes and challenges (leading to slippage)
- Lessons learnt during the implementation
- Positives and negatives of certification
- Sanitation entrepreneurs and marketing strategies
- Involvement of people with disabilities and the elderly in ODF and HWWS activities
- Priorities moving forward (what to be removed and what to be incorporated in the new strategy)

Data collated through KIIs was documented as notes and summarized for triangulation with findings from the desk review and FGDs.

Table 3: Targeted individuals for KIIs (n=24

District Level	District/Department	KIIs	ODF Status
North	Rumphi	4	ODF with slippage
	Mzimba South	5	Recently declared ODF
Central	Nkhotakota	3	Recently declared ODF
	Mchinji	4	Not ODF
South	Balaka	4	ODF with slippage
	Mwanza	4	Not ODF

Key Informants (n=24): These included WASH coordinators, DEHOs, SHIN coordinators, District Directors of Water, Developmental Partners of WASH including the following: World Vision, Feed the Children, United Purpose, Project Concern International, Malawi Red Cross, ONSE, NAYORG, CADECOM, Participatory Rural Development Organization (PRDO), Water Aid, Plan International, Hygiene Village, Synod of Livingstonia Development (SODEV).

Data from these interviews was consolidated and summarized as both qualitative and quantitative data. Saturation was achieved in responses from stakeholders in all sectors (implementing partners, district staff, donors, government, etc.).

3.2.3.2 Focus Group Discussions

Focus group discussions were conducted to provide feedback and insight from the beneficiaries in communities involved in the implementation of the ODF and HWWS strategies. FGDs were designed to allow for open discussion with appropriate delineation by gender, position and role in the implementation and achievement of ODF status (Table 4).

Table 4: Target populations for FGDs

Target group	Definitions
Males	Person primarily responsible for ensuring the availability of sanitation and hygiene facilities in the household
Females	Persons primarily responsible for ensuring the availability of water in the household, cleaning of sanitary facilities, supervising young children when using the toilet and washing their hands
Youths	Usually target beneficiaries and change agents in school sanitation and hygiene initiatives
Vulnerable populations	People with disabilities and the elderly
Hygiene Promoters (i.e. natural leaders and/or TAs and HSAs)	Those that promote behavior change, provide low cost latrine construction advise and hygiene education
Local leaders and teachers	Local leaders and teachers will be especially those trained as facilitators to implement ODF and HWWS activities in their respective villages and schools
Sanitation entrepreneurs	Enterprises that play roles in the provision of sanitation products and services in the communities

Overall 6 Districts were targeted (2 in each region) to provide an array of ODF status including (1) recently declared ODF, (2) achieved ODF but now showing slippage and, (3) not declared ODF. In each district the team attempted to conduct 6 FGDs as outlined in Table 5.

Each FGD aimed to have 6 – 10 participants, and purposive sampling was used to recruit participants of the FGDs. Each FGD followed a guide (Appendix 2) and addressed the following key areas:

- Availability and use of sanitation and hygiene facilities
- Knowledge, drivers, motivators and barriers of hand washing behavior
- Disposal practices for children's feces
- Open defecation
- ODF and HWWS slippage

Table 5: Outline of planned FGD programme

Region	District	FGDs	ODF Status
North	Rumphi	In each District:<br>1 x men<br>1 x women<br>1 x leaders<br>1 x marginalized<br>1 x youth<br>1 x entrepreneurs<br>1 x health promoters	ODF with slippage
	Mzimba South		Recently declared ODF
Central	Nkhotakota		Recently declared ODF
	Mchinji		Not ODF
South	Balaka		ODF with slippage
	Mwanza		Not ODF

FGDs were recorded using digital recorders and were summarized in notes to allow isolation of themes.

In total the following FGDs were achieved:

- Local leaders n=6
- Sanitation promoters n=6
- Males n=4
- Females n=6
- Youths n=6
- Marginalized n=6
- Entrepreneurs / Masons n=4

Saturation in responses was achieved by the completion of these FGDs.

3.3 Consolidation of data

Following the collection of all data through these 3 key sources:

- Desk review (including feedback from National forums)
- Key Informant Interviews
- Focus Group Discussions

The information was consolidated under specific thematic areas relevant to each the ODF and HWWS strategies. Initial key findings were presented to the National stakeholder ODF review in November 2017, where they received comment, validation and supplementary information. This has been included within this report.

The Findings section of this report therefore represents the outcomes of all stages of evaluation.

4.0 FINDINGS

As described earlier, findings are outlined under specific objectives, which provide a thorough exploration of the ODF and HWWS strategy's successes, barriers and challenges in the last 6 years. These findings are drawn from a combination of desk review, KIIs, FGDs and stakeholder analysis.

4.1 Effectiveness of the scope, mechanisms and actions applied in the implementation of the ODF/HWWS Strategies

4.1.1 Scope, mechanism and action in current strategies

Strategy		
ODF Strategy	**Scope:**	Achieve Open Defecation Free (ODF) status for rural communities and schools in Malawi by 2015
	**Mechanisms:**	Community led total sanitation and sanitation marketing with oversight from the National Open Defaecation Task Force
	**Actions:**	Training and capacity building of HSAs, Traditional Leaders, Natural Leaders and Masons<br>CLTS process: Triggering, Verification, Certification<br>Supporting actions: Traditional leader support, publicity, networking, coordination and integration
HWWS Strategy	**Scope:**	Accelerate the adoption of hand washing with soap behaviour in Malawi
	**Mechanisms:**	Communications, pubic private partnerships, social marketing and participatory approaches
	**Actions:**	Making hand washing facilities available<br>Using health facilities and schools as models and agents of change<br>Behaviour change communications<br>Supporting actions: Integration

Ultimately the effectiveness of the scope of the strategies is based on the impact they have achieved since their implementation in 2011. The prevalence of OD in 2011, (National ODF Malawi 2011-2015 Strategy) was estimated at 11%; and in 2017 (JMP, 2017), is now estimated at 6%, demonstrating significant reduction. In terms of HWWS, although progress has been reported, there is still a significant way to go to ensure HWWS is possible and being undertaken at critical times. An increase of 10% from 24 – 34% coverage of hand washing has been reported between 2011 and 2016. However, this does not necessarily reflect the presence or use of soap.

The figures do demonstrate that from 2011-2017, there have been positive results and progress towards strategic targets (DeGabrile and Ngwale 2017). Nevertheless there are still significant barriers and challenges to the achievement of the key goals of ODF and HWWS across Malawi.

"Although steady progress has been made in triggering communities - overall, 80% of villages have been triggered and 70% have achieved level 1 ODF - many districts, particularly those struggling with co-resourcing with partners, are still lagging behind. Besides making good strides in achieving level 1 ODF in communities, sanitation is lagging behind in institutions such as schools, hospitals, prisons and in public places such as markets and trading centres where pit-latrines are in short supply. Furthermore, nationwide, hand washing facilities are in short supply and/or not available in both communities (including those that achieved ODF) and institutions. Consequently, the behavior of hand washing with soap remains a challenge"- ***Summary from District Environmental Health Officers and District Water Officers from visited districts***

4.1.2 Key findings

Scope:

- Both the ODF and HWWS strategies do not provide specific definitions of latrines, hand washing facilities, etc. which leads to variation in implementation.
- The ODF strategy focuses only on the rural population, which has limited the focus and success of ODF achievement.
- ODF strategy implementation has focused on households with limited improvements in schools, and no action taken in other institutional settings. For example, declared areas have schools, prisons, markets and health facilities which do not have sufficient facilities and show evidence of open defaecation. During our survey, we found that most schools had basic latrines (i.e. with muddy floors and grass thatched) and often times the latrines were inadequate so that the pupils resorted to using the bush.
- There is no reference or integration of ODF strategy with menstrual hygiene management.
- Neither strategy has specific reference or support for vulnerable and marginalized groups.
- The ODF strategy does not consider the whole sanitation chain (capture to disposal).

Mechanisms and Actions:

- ODF strategy implementation was to be overseen by the National Open Defaecation Task Force (NOTF) which represents the Ministry of Health and the Ministry of Agriculture, Irrigation and Water Development with key development partners and civil society.
- The current ODF strategy is limited to the use of CLTS and sanitation marketing and does not take into consideration the use of other participatory approaches such as PHAST and mechanisms to achieve ODF.
- Although there is the inclusion of 2 levels of ODF status, there is little reference to the effective use of the sanitation ladder to achieve continued improvement and sustainability.

- The HWWS strategy is limited in terms of integration with other key strategies (e.g. Nutrition, mother and child health, immunisations, school health and nutrition, etc.) and relies largely on mass media rather than focused behavior change communication.
- Both strategies lack reference to specific standards and guides for the construction of toilets and hand washing facilities.
- HWWS strategy uses health facilities and schools as key locations for good practice and development of agents of change, but in many cases these were identified as having the poorest standards.

4.1.3 Key recommendations

Scope:

- The scope of the ODF strategy should include the following:
    - Definition of a latrine (development of associated standards).
    - Consideration of the whole sanitation chain.
    - Both rural and urban areas to be targeted.
    - Inclusion of institutions and public settings, i.e. hospitals, schools, health facilities, markets, prisons, offices, etc.
    - Specific reference to the inclusion of vulnerable and marginalized groups
    - Integration of menstrual hygiene management standards and considerations particularly in institutional and public settings.
- The scope of the HWWS strategy is wide ranging and does not require any specific changes

Mechanisms and Actions:

- NOTF should be more multidisciplinary in its membership with the inclusion of representatives from Nutrition, disabilities and other appropriate government departments to ensure integration of services.
- The mechanisms and actions of the ODF strategy should consider:
    - A requirement of all settings to meet targets for ODF before an area can be declared ODF. These standards should be integrated with those of other Ministries and WHO/UNICEF (e.g. school standards in terms of numbers and condition/cleanliness).
    - The inclusion of other mechanisms and participatory approaches to achieve ODF.
    - Inclusion and use of the sanitation ladder as a strategy for communities to move from Level 1 to Level 2 ODF status. For example the use of PHAST approach will assist communities understand their problems, plan and implement activities that will lead to improved sanitation and promote hand washing with soap?
    - Effective integration with a wider range of strategies
    - Provision of standards and specifications to guide ODF implementation.
- The mechanisms and actions of the HWWS strategy should consider
    - Effective integration with a wider range of strategies
    - Provision of standards and specifications to guide HWWS implementation.
    - Ensure the sites for good practice are able to achieve these standards.
    - Improve focus on private sector engagement and public private partnerships to help provide effective hand washing facilities, affordable soaps, etc.

4.2 Review of current country strategies with emphasis on original assumptions

4.2.1 Open Defaecation Free Malawi Strategy 2011 – 2015

The ODF strategy was developed with a range of CLTS stakeholders in March 2011. At this time they agreed on CLTS as a key strategy for Malawi to achieve ODF by 2015, but recognised the need for the inclusion of other issues to complement CLTS including sanitation marketing, involvement of traditional leaders, publicity of the strategy, mobilization of resources and better coordination. As such the strategy components were outlined as depicted in Figure 12.

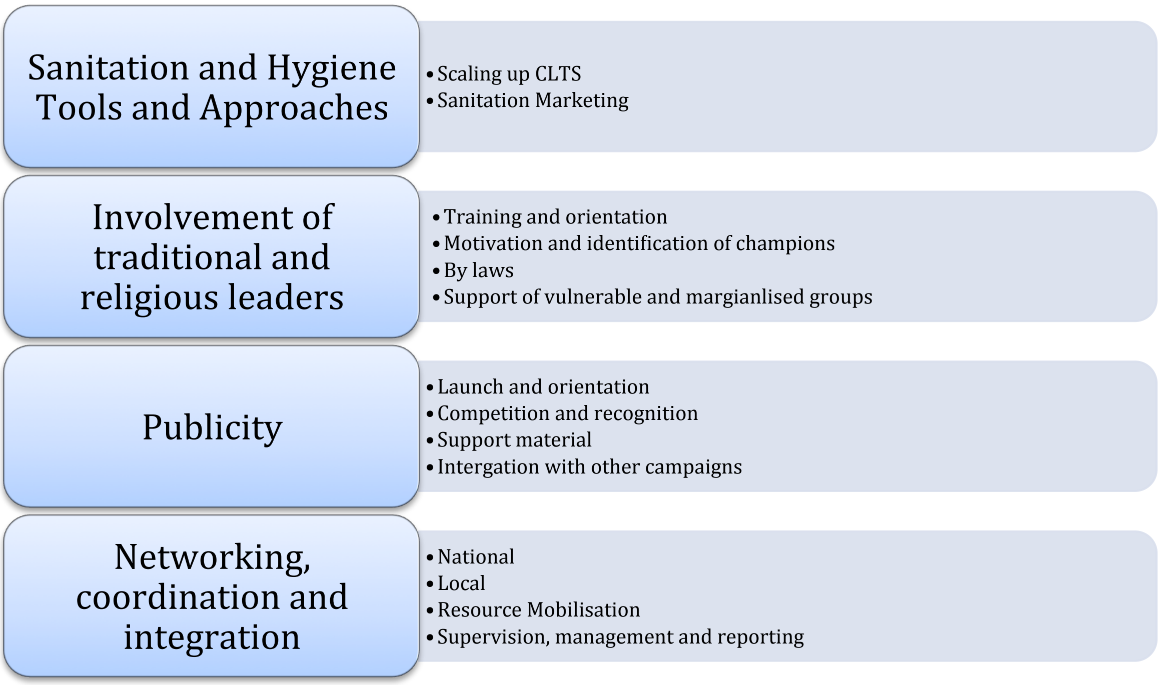

Figure 12: Outline of the main components of the ODF Malawi Strategy 2011 - 2015

4.2.1.1 Component 1: Sanitation and Hygiene Tools and Practices

Key Assumptions

During the development of the strategy, key assumptions were made on the achievement of rapid ODF status for Malawi:

- CLTS had been shown to be effective in 12 districts with 40% of triggered villages achieving ODF status within 2 years of introduction. As such CLTS was deemed to be the most effective tool for achieving ODF over the 5 year strategy and outlined as the only method to be adopted for scaling up.
- Lessons learned from CLTS introduction since 2008 indicated that sanitation marketing could support communities in the achievement of effective ODF and to climb the sanitation ladder through locally available sanitation masons, suitable low cost technologies and systems of financing. These were to be developed in conjunction with CLTS triggering.
- District and traditional authority level teams would be trained and would then operate to expedite the process of triggering, monitoring and verification.
- HSAs would absorb CLTS and ODF activities into their routine activities as part of their role in sanitation and hygiene. This would include reporting of village level data.
- Natural leaders would be identified at village level and would support the achievement of ODF status.
- School Led Total Sanitation (SLTS) was deemed to be an effective approach to achieving ODF schools and supporting communities to achieve the same.
- ODF verification would be an open and transparent process which would only reflect those communities which have truly achieved ODF through infrastructure and behaviour change
- Achievement of ODF Level 1 would automatically lead to a community striving maintain the status and/or to achieve Level 2.

Key findings and Recommendations

A review of progress against these assumptions highlighted the following key findings and recommendations:

CLTS as a focal methodology

According to Chambers, participatory rural appraisal enables local people to share, enhance and analyse their knowledge of life and conditions, to plan and to act' (Chambers 2009). CLTS applies the principles of participatory rural appraisal to facilitate the community to analyse the problems associated with open defecation and to trigger all members of a community to construct a household latrine (Chambers 2009).

For example, CLTS was adopted by the Government of Kenya as a national sanitation strategy in 2011 following successful piloting by sector players since 2007. Significantly, between 2010 and 2011 this initiative registered impressive results with over 1,000 villages (571,231 people) attaining open defecation free status. Consequently, in May 2011, the Government and partners launched the ODF Rural Kenya 2013, campaign which aims to eradicate Open Defecation (OD) in Rural Kenya by 2013. (ODF rural Kenya, 2013)

The ODF success rate, defined as the proportion of triggered communities that become ODF, is a key indicator of the effectiveness of CLTS implementation. The ODF success rate does not tell us anything about the quality or sustainability of collective sanitation outcomes, but it is a key indicator of CLTS effectiveness that can highlight problems as programmes spread and scale up.
The CLTS approach focuses on triggering rural households to use locally available resources to build and use affordable latrines that meet their sanitation needs.

It should be noted that the ODF success rate might be expected to decrease as programmes scale up due to the more difficult physical conditions and challenging social contexts encountered, and the challenges of maintaining the quality of CLTS facilitation and processes on a larger scale. In practice, most CLTS reviews find substantial variations in ODF success rate across both large and small programmes, and even under the same conditions within the same programme (**UNICEF, 2013).** The country CLTS overviews suggest that government sanitation policy and technical guidelines are important factors in the scaling up and effectiveness of CLTS programmes. CLTS uses a number of different mechanisms to encourage sanitation behaviour change among the poorest and most disadvantaged households, including disgust, peer pressure and collective action. **(UNICEF, 2013)**

Recommendations

- ***Improve CLTS enabling environment:*** *Scaling up CLTS progress and improving CLTS effectiveness and sustainability will be dependent on further strengthening of enabling environments for rural sanitation. In Malawi, governments or development partners in the region have yet to develop strategic sanitation plans that elaborate the role of CLTS in creating large-scale demand for sanitation, or financed national implementation programmes that combine CLTS with other approaches. Realistic, costed and well prioritized strategic sanitation plans are central to persuading governments, which historically prefer infrastructure investments that it is in their interest to allocate more finance and capacity to behaviour-change programmes like CLTS which can reach the poor and reduce health costs (UNICEF, 2013).*
- ***Strengthen CLTS monitoring systems:*** *There is need for more detailed monitoring and evaluation of CLTS progress and effectiveness. There is a need for more regular updating and reporting of national CLTS and other sanitation progress data. Annual strategic reviews, ideally linked to the monitoring of CLTS progress against strategic sanitation targets, and local government benchmarking systems are useful mechanisms for pulling monitoring data and reports up through government and programme systems. In particular, the strategy reviews must consider recommendations that allow the streamlining of M&E in the ODF sector both for infrastructure and behavior change and should consider guidance and recommendations of the World Bank* Innovations in WASH Impact Measures: *Water and Sanitation Measurement Technologies and Practices to Inform the Sustainable Development Goals (2018). This will also support the alignment of indicators with the SDGs to provide a basis for progress measurement.*

- ***ODF sustainability*** *Demand for information on ODF sustainability is essential and, therefore, we support the recommendations on ODF sustainability highlighted in DeGabriele and Ngwale study (2017). Despite frequent suggestions by the stakeholders*

at the annual review meetings that follow-up and long-term support after CLTS triggering are critical to sustainability, there has been little finance or capacity allocated to these areas by projects or programmes. We therefore further recommend that greater priority be allocated to post-triggering activities in plans, programmes and practice, and that efforts should be made to document best practices for the long-term institutional support and monitoring of ODF and non-ODF communities. Concerns about the possible negative effects of institutional incentives for collective sanitation improvement on sustainability should be addressed (information gathered during FDGs revealed that some chiefs monopolized the incentives such as plates and basins. They kept these in their houses and never shared with the communities). Nonetheless, the broad family of incentives available, which include numerous non-financial awards in addition to more conventional conditional grants and financial rewards, offers a useful mechanism through which to increase the monitoring and support provided to post-ODF communities.

- ***Latrine hardware subsidies.*** *Our desk review and field data indicate that a policy on latrine hardware subsidies remains an emotive and important issue. More effort is required to understand how government and development partner policies on latrine hardware subsidies can be improved (The discussions held at the annual review meeting of November, 2017 highlighted the strong polarity of those that either support or oppose latrine hardware subsidies) and better aligned with CLTS, sanitation marketing and other interventions designed to improve rural sanitation and hygiene. At the very same annual review, donor agencies strongly felt that subsidies should only be applied to the vulnerable or marginalized (disabled).*

Sanitation marketing as a means to achieving ODF

The use of sanitation marketing in conjunction with CLTS was a logical progression at the time of the strategy development, as this would support the movement of households up the sanitation ladder through the availability of local skilled labour, and would create income opportunities for local masons, etc. However, feedback from the range of stakeholders indicated the following challenges and missed opportunities:

- Focus of many partner organisations has been on the training of masons.
- Despite training of masons and entrepreneurs, there has been little demand created for services in target communities.
- Households appear to be constructing poor quality latrines which are not sustained and collapse. Therefore there is a need for improved construction and use of masons however this is limited by the financial capability of the households and the pressure to construct.
- Once a community has been declared ODF, there is currently little progress to ODF++ (primarily attributed to economic challenges) and as such the need for masons and entrepreneurs is limited.

Sanitation marketing was intended to be working in conjunction with community based financing systems, thereby addressing this issue of economic challenge, and removing the need for subsidies which were deemed unsustainable. This approach has been successful elsewhere, for example evidence from Cambodia shifted focus from subsidy to a market-based approach and achieved an increase in sanitation facilities through the use of a

market-model. In this case, families are working in groups and contribute monthly to provide toilets to households. Together they pay monthly installments and on rotation, one family receives a toilet each month until all the households in the group have received a toilet (Phyrum et al. 2012).

Feedback from the range of stakeholders indicated the following challenges with financing systems under the current ODF Malawi strategy:

- A number of partners have tried specific community financing systems with varying rates of success. It must be considered that village based saving and loans schemes are now ubiquitous in rural settings, and these have worked very successfully. However, the use of funds from these schemes may be not being focused on the construction of sanitation and hygiene facilities but rather school fees, etc. With this in mind, the system itself may work effectively, but the use of funds may be prioritized differently in the community's perspective.
- Numerous development partners and government implementers requested consideration of the reinstatement of subsidies.

Recommendations

- *Effective sanitation marketing and financing models need to be more fully integrated into CLTS triggering programmes.*
- *There should be an emphasis on the role of sanitation marketing and supportive financing to assist movement of communities from ODF to ODF++*
- *As outlined above there is a need to have a standardized compendium of sanitation and hygiene options, which are approved and suitable for specific environments.*
- *Movement towards a requirement for standard systems to be constructed should be considered which would improve quality of latrines and create business for masons and entrepreneurs.*
- *Training of masons should be linked to technical training colleges and schools.*
- *Further research and understanding of tested financing systems, and the prioritization of household income with relation to sanitation and hygiene expenditure is needed.*

Efficacy of District and Traditional Authority teams and use of Natural Leaders

In order to accelerate implementation the Strategy required the development of TA level teams who would identify and incorporate Natural Leaders to work with District extension staff. This element complements the Strategy Component 2 – Involvement of Traditional and Natural Leaders. As such full feedback on those roles is outlined within that section. However it was also noted by partners that the aim of the TA team was to provide multidisciplinary support to achieving ODF. However in the majority of cases the responsibility for CLTS had landed specifically with the HSA in the area, with limited input from other extension workers.

Recommendations

- *The implementation, achievement and maintenance of ODF status needs to be supported by a multidisciplinary team.*
- *The ODF team should integrate with the existing community structures and reflect those now agreed and adopted through the National Community Health Strategy (2017 –*

2022). As such, community members should be involved through the Prioritised Community Structures (Figure 2a) specifically the Village Health Committee and Community Health Action Group in conjunction with the District staff in the Community Health Team. (Figure 13)

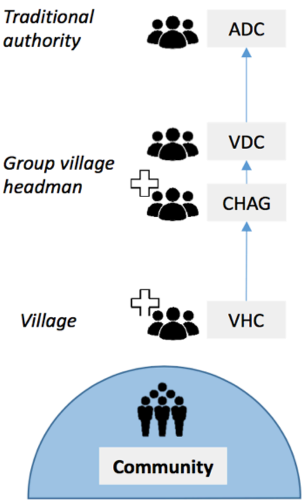

Figure 2a: Prioritised Community Structures (NCHS 2017)

(VHC: Village health committee; CHAG: Community Health Action Group; VDC: Village Development Committee; ADC: Area Development Committee)

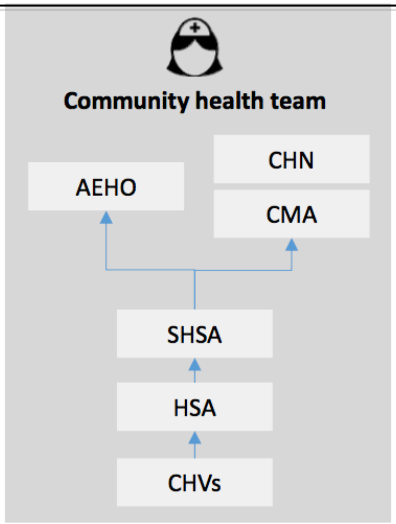

Figure 2b: Community Health Team

(CHVs: Community Health Volunteers; HSA: Health Surveillance Assistants; SHSA: Senior Health Surveillance Assistant; CMA: Community Midwife Assistant; AEHO: Area Environmental Health Officer; CHN: Community Health Nurse.

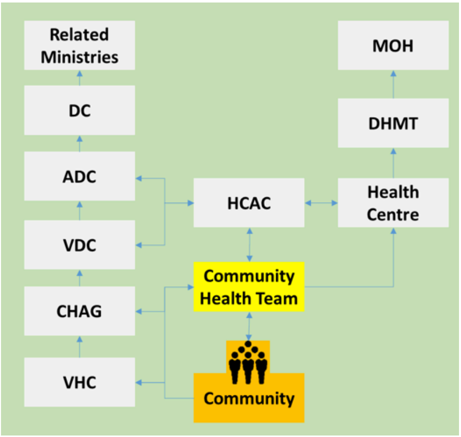

Figure 2c: Community Health Systems Structure

(VHC: village health committee; CHAG: Community Health Action Group; VDC: Village Development Committee; ADC: Area Development Committee; DC: District Council; HCAC: Health Centre Advisory Committee; DHMT: District Health Management Team; MOH: Ministry of Health)

Figure13: Community Health Structures

HSA implementation through routine activities

As stated above, there was a specific emphasis on the role of HSAs in the delivery of CLTS and subsequent achievement of ODF. However, partners and implementers indicated the following challenges in this area:

- The use of HSAs in the drive for ODF was reported as inconsistent across partners.
- CLTS was seen as a 'project' by HSAs, and once partners were gone the implementation also stopped.
- HSAs were used to receiving allowances to undertake this work and therefore stopped their CLTS/ODF activities when they became routine activities.
- Data was inconsistently reported and in some cases validity is called into question.
- HSAs in a number of areas were reported to be using village health committees and volunteers to undertake their roles in ODF attainment.

Recommendations

- *ODF must be incorporated into the routine activities of HSAs without the requirement of allowances.*
- *There should be reference to the Role Clarity Guidelines produced by the Ministry of Health in 2017 for clear roles of HSAs, VHCs and other interested parties.*
- *Funding must be ring-fenced for ODF activities from the District budget.*
- *ODF activities should be integrated with other work to make best use of time and funds. This will be support by the Integrated Service Guidelines to be produced for community health workers in 2018.*

SLTS as a tool to promote ODF

Despite the intention to use schools as a platform to promote SLTS and subsequently CLTS, it was reported that youth had not been actively involved in village sanitation and hygiene. Nevertheless it was agreed that this should be a consideration when moving forward to improve youth participation and integration of ODF messaging.

Recommendations

- *Issues of sanitation and hygiene including the necessity for open defaecation free environments must be clearly included in both primary and secondary school curricula.*
- *Schools must be required to meet minimum standards before an area can be declared open defaecation free.*
- *School Water, Sanitation and Hygiene standards and guidelines require be finalizing and integrating with the updated ODF strategy.*
- *SLTS should begin at Early Childhood Development Centres (previously CBCCs).*

4.2.1.2 Component 2: Involvement of Traditional and Religious Leaders to accelerate progress at the community level

Key Assumptions

It was assumed that increased involvement by traditional and religious leaders was seen to have great potential in enabling Government to reach the goal of attaining an ODF Malawi by 2015. Based on this principle the following additional assumptions were made:

- All traditional and religious leaders would be trained and orientated in the requirements of the ODF Strategy
- Leaders would be willing to support and motivate their community members to achieve ODF.
- ODF Champions would be identified in leaders who would promote the cause of ODF nationwide
- Leaders would be willing to set up and enforce by-laws to achieve and sustain ODF status in their areas.
- Leaders would interact regularly with district staff to discuss and support ODF achievement
- Leaders would identify vulnerable households and ensure community support for the construction of latrines etc.

Key findings and Recommendations

- As outlined above, the TA was recognized as a key level at which teams should be operating simultaneously to achieve accelerated implementation of CLTS and subsequent ODF status. It was reported that these teams have been more effective in some TAs than others, and where leaders and TAs were not supportive of this programme there has been little progress.
- Community members and partners indicated that in some areas households are not motivated to change as they see leadership with no latrines.
- Numerous TAs have utilized by-laws as a means to enforce latrine construction and have reported success on this basis. However there were conflicting issues raised by respondents:
    - Respondents indicated that by-laws had supported the rapid achievement of ODF in some areas. For example *"Strong local leadership (sanctions / by-laws) have led to achievement of ODF", (*Respondent, Mwanza FGDs).
    - Community members reported that they feel the sanitation improvements are 'imposed' on them and do not necessarily lead to use or sustainability even if the toilet is in place.
    - Leadership had been reported to give preferential treatment to friends and family when they impose by-laws.
    - Concerns were raised that the use of by-laws negated the real implementation of behavior change communication which would affect sustainability and willingness to scale up to ODF++
- Stakeholders reported the need for integration in community structures for effective implementation, and the valuable role of Natural Leaders.
- There was little reference from any stakeholder on the use or support of religious leaders in achieving ODF.

- Vulnerable and marginalized groups were supported in some areas, however it was felt that they should be engaged from the offset of the CLTS programme and be involved in the training, implementation and verification processes to ensure appropriate systems are in place to support them.
- Specific designs should be provided for suitable latrines for those with disabilities to ensure safe use and dignity.

Recommendations

- *Continued use of traditional and natural leaders to support the implementation, achievement and sustainability of ODF status.*
- *Formal recognition of natural leaders and their roles in community sanitation and hygiene achievement.*
- *Effective integration of leadership (traditional and natural) activities with community structures.*
- *By laws should continue to be encouraged but must be enforced consistently for all community members.*
- *By laws should not be seen as the main solution but must be supported with effective behavior change communication strategies to achieve sustained ODF environments.*

4.2.1.3 Component 3.0: Publicity

Key assumptions

The ODF 2011 – 2015 strategy recognized that publicity would need to play a key role in the achievement of ODF status based on a number of assumptions:

- Buy-in from central government, district government, politicians, and traditional leaders to ensure national support.
- Willingness of TAs to engage in competition.
- Willingness of the media to publicise and promote ODF achievements.
- Finances to support recognition ceremonies and willingness of high profile persons to attend and support celebrations.
- Effective coordination with other publicity campaigns to achieve integration.

Key findings

The majority of feedback regarding publicity pertained to the celebrations and ceremonies which take place when ODF is achieved at TA level.

- With regard to the awards given, results from FGDs and KII as well as stakeholders analysis revealed that giving was a good way of incentivizing the community. However, respondents noted that some Traditional leaders would keep the gifts (especially plastic basins and cups) for personal use.
- Although motivating, there is a high cost associated with the ODF celebrations that take place at TA level and the cost – benefit of these should be considered.
- Participants stressed the lack of progress after the ceremonies and celebrations have passed and the progressive slippage which then occurs back to OD.

- Integration of the ODF strategy with others such as HWWS was not achieved effectively.
- Integration of communication on ODF with other strategies was also limited.

Recommendations

- *Communities need to sustain their ODF status and progress up the sanitation ladder. To support this, after they have been declared ODF additional activities should be added e.g. competitions and rewards, whereby households which are exemplary should be rewarded.*
- *Large ODF celebrations attended by the Minister and dignitaries should only take place when the District has achieved ODF status.*
- *Consideration should be given to the integration of the ODF and HWWS strategies to one strategy in the future. It must be borne in mind that these strategies can also benefit from stand-alone status which allows their easy integration with other strategies such as water, maternal health, nutrition, community health, etc.*
- *Effective use of Water and Environmental Sanitation Network (WESNET) to coordinate between national publicity campaigns to ensure message integration and best use of funds.*

4.2.1.4 Component 4.0: Networking, Coordination and Integration

Key Assumptions

- National coordination and local level coordination channels
- Resource mobilization and leverage by various stakeholder and government
- Communication between national and local level
- Information management and reporting
- Supervision
- Effective use of subsidies

Key findings

- Documentation of the success, failure, or lessons to be learned from CLTS and ODF achievements is haphazard. Naturally, there is an inclination for those involved in the innovation (either through its implementation or its funding) to claim success for it. From the documents obtained in the districts, it has been shown that there was little comparative documentation of what worked and what did not on a programmatic basis in order to determine whether or not the projects were in fact replicated, or creatively adapted, for expansion (Dutton, et al, 2011). This review can help to identifying where and why they have failed or succeeded and the future route to be taken.
- Results from the FDGs and KIIs indicated that ODF achievement was attributed to support and zeal from partners, multi-sectoral collaboration and better coordination. One of the participants from the KIIs echoed that "*Proper coordination works wonders".*
- NOTF engaged partners well but government has not improved funding of preventive WASH activities.
- The WASH sector in Malawi has vast actors but WESNET echoes that there was minimal coordination and knowledge sharing of efforts to drive a concerted agenda.

- There were very minimal efforts towards networking and learning in the WASH sub sector in Malawi.
- Fragmented work by the WASH actors in Malawi is perpetuated because of a gap in the understanding on what each actor is doing.

Recommendations

- *Participants felt that there is need to "strengthen stakeholder collaboration in both CLTS and SLTS".*
- *WESNET emphasized that there is need for Government to prioritise WASH and not treatment of WASH related diseases where almost MK8 billion is spent yearly unlike in 0.03% of the ORT funds being sub vented to WASH activities.*
- *More effective use of WASH NGO and donor groups for shared learning and planning.*
- *Circulation and maintenance of the national WASH directory created by WESNET so that there is a clear picture on what each actor in WASH is doing for easy coordination and knowledge sharing (WESNET directory, 2017).*

4.2.2 Hand Washing With Soap Campaign (Strategy) 2011 – 2012

The HWWS Campaign 2011 – 2012 for Malawi was developed in consultation with key Ministries and partners. The overall objective was to_accelerate the adoption of HWWS behaviour in Malawi. The Campaign adopted 8 strategies to achieve this as outlined in Figure X.

Development of key messages	• Targeting critical times for handwashing
Integration of HWWS with CLTS	• Integration of HWWS promotion with CLTS triggering to achieve ODF++ status
Ensuring availability of hand washing facilities	• Development and promotion of suitable HWF<br>• Use of social marketing to promote use
Use of social marketing and participatory methods	• Understand the behaviours to target in communities<br>• Use of PHAST as a particpatory tool
Use of school children as agents of change	• Linked with SLTS<br>• Teachers to combine HWWS promotion with lessons and activities
Use of health facilities as models for HWWS	• Good practice modeled by health workers<br>• Health promotion showing model HWF and practices
Use of multiple channels for behaviour change	• Use of mass media and partners to promote HWWS messages
Public Private Partnerships	• Develop partnerships and ensure consistent messages from private sector<br>• Harmonisation of messages from government, NGOs and private sector

Figure14: Summary of key strategies within the HWWS Campaign 2011 -2012

4.2.2.1 Development of Key Messages

Key Assumptions

The assumptions made were that the campaign will promote hand washing with soap at critical times with specific emphasis on:

a. Before preparing food (particularly for infants and breastfeeding) and eating
b. After visiting the toilet and cleaning a baby's bottom

Key findings

Respondents indicated that the majority of households are aware of the key times when they should wash hands but there are still beliefs which affect the uptake of hand washing with soap.

- Households wash hands with water only and do not see the value of using soap to improve this.

- Child stools (particularly those from 0 – 6 months) do not contain bacteria which can cause illness.
- Hand washing facilities are not conducive to supporting effective and easy hand washing.
- Use of soap is prioritized for other household activities such as bathing, washing clothes, etc.
- When left at hand washing facilities soap is often stolen, or eaten by an animal which wastes a precious resource.

Recommendations

- *Messaging must be more specific and combined with the behavior change communication elements to target factors more likely to achieve change.*
- *More effective hardware needs to be developed to encourage HW and HWWS.*

4.2.2.2 Integrating HWWS promotion in Community Led Total Sanitation Community Led Total Sanitation (CLTS).

Key Assumptions

This strategy assumed the effective implementation of the ODF Strategy 2011 – 2015, and with that the effective messaging of HWWS to be combined with sanitation triggering using CLTS methods.

Key findings

- CLTS triggering focused on the construction and use of latrines with less emphasis on the need for HWWS.
- The use of disgust as a trigger for HWWS may not be an effective one and therefore not encourage practice.
- The ODF strategy only required the presence of a hand washing facility and soap to achieve Level 2 certification. To date communities have primarily achieved ODF at Level 1 which does not have a requirement for a hand washing facility and therefore there has been less emphasis on this practice.

Recommendations

- *Emphasis of the need for effective hand washing with soap in combination with sanitation facilities to be strengthened.*
- *Improved integration of strategies and campaigns during implementation.*

4.2.2.3 Ensuring availability of hand washing facilities

Key Assumptions

- The campaign would drive the design and development of low cost effective hand washing facilities, which could be adopted at institutional and household level.

Key Findings

- There is still a significant issue with the development and use of appropriate HWF in Malawi both at household at institutional level.
- Schools do not have suitable facilities to ensure effective HW or HWWS for all students.
- Households may have facilities but lack of access to water can mean they do not prioritise use for hand washing.
- HWF used at the moment are not durable or sustainable as they are damaged by animals and damaged by the sun after a few months exposure.
- Placement of soap at HWF is frowned upon as it is often stolen or eaten by animals.
- There is primarily an emphasis on HWWS for rural communities only without consideration to urban, institutional and workplace settings.

Recommendations

- *A range of effective, low cost and sustainable HWF is needed for households and institutions to guide them on good practice.*
- *School WASH standards need to be completed and circulated to ensure improvement at facilities. These standards must include a range of low cost HWF suitable for school settings.*
- *The requirement of HWF must be set across all platforms – home, workplace, school, health facilities, prisons, etc.*

4.2.2.4 Use of social marketing and other Participatory approaches

Key Assumptions

This campaign assumed the use of:

- Social marketing of HWWS support materials, e.g. soaps, facilities, etc. through district councils, NGO partners and private sector
- Participatory approaches such as PHAST to promote HWWS behaviours.

Key Findings

- Social marketing of HWWS materials was minimal and based on small pilot testing of materials and systems with little learning shared in the WASH sector for potential scale up.
- The use of PHAST to promote hand washing was contradictory to the use of disgust through the CLTS approach. This may have led to confusion in facilitators on the approach to be used. This would also have been impacted by the greater emphasis on ODF achievement without HWWS.

Recommendations

- *Sharing of findings from programmes which have promoted HWWS successfully through HWF and soap provision through social marketing channels*
- *More emphasis should be placed on social marketing through coordinated efforts with the same messages to ensure consistency and reduce confusion for beneficiaries and target audience.*

- *Ensure the consistency in approaches and integration of their implementation to reduce replication, and best use of funds. This should be through the appropriate community health channels (Figure 2a).*

4.2.2.5 Use of School Children as agents of change

Key Assumptions

- School children (including CBCCs) can provide an eager and willing resource that can be instrumental in bringing messages home, to friends/peers, family and relatives.
- Teachers would combine campaign activities with school lessons (e.g. poster competitions in art classes) and promote devices for facilitating hygiene behaviour that are appropriate for both school and homes.
- The campaign would deliberately link up with sanitation promotional activities such as School WASH and CLTS as a way of providing sanitary facilities.

Key findings

- Little progress has been made in sanitation and hygiene practices in schools which are still underserviced.
- Current systems being put into schools for HW are not appropriate for the quick throughput of high numbers of students.
- Schools do not provide soap for students to use due to cost, theft, etc.
- Teachers are not integrating issues of HWWS into their day to day curriculum.
- There are currently no standard guidelines for sanitation and hygiene requirements at schools.
- Parents and other adults do not always value the opinion of children when they come home with hygiene messages *"you can do that at school but you do not need to do that at home"*

Recommendations

- *The concept of using schools and children as agents is still a welcome one but needs better integration and structure:*
    - *HSAs to be more involved at schools for sanitation and hygiene advice and support*
    - *School WASH guidelines to be completed and implemented to set specific standards.*
    - *Schools to be provided with a range of low cost options for HWF which are appropriate of high number of students. This includes the use of soap and protection against theft.*
- *HWWS needs to be better linked with the proposed SLTS programme.*

4.2.2.6 Use of Health Facilities models for hand washing with soap

Key Assumptions

- Good practice at health facilities should promote good practice at home.

- Health workers can use the opportunity at clinics to promote good HWWS practice and demonstrate HWF options

Key Findings

- Most health facilities and other institutions in the ODF declared areas had no HWWS facilities.
- Health facilities and health workers were not promoting effective hygiene behaviours during clinics due to lack of facilities.

Recommendations

- *Certification of areas and TAs for ODF should also take into consideration the availability of HWWS facilities in these private institutions.*
- *Health facilities must be supported to ensure that they are modeling improved sanitation and HWWS to promote good behaviour.*
- *HWWS promotion needs to be integrated into all relevant clinics, e.g. antenatal, growth monitoring, immunisations, OPD, etc.*

4.2.2.7 Use of Multiple Communication Channels for Behaviour Change

Key Assumptions

- An effective mix of communication channels would be used from mass media, to direct contact.
- Institutional settings such as churches, health facilities and schools would facilitate behaviour change communication.

Key Findings

- Behaviour change messages were generalized and were not always based on sound research and understanding of how these would influence practice.
- Messages through different channels were not consistent and as such caused confusion.
- Institutional settings were not using good practice and were therefore not communicating effective behaviour change messages.

Recommendations

- *Behaviour change messaging needs to be developed based on sound principles and with an understanding of the audience and behavioural factors which are being targeted*
- *"Teachable moments' must be identified to maximize impact of messaging.*
- *Settings for messaging must be demonstrating good practice.*
- *Messages must be consistent and coordinated to minimize confusion and achieve maximum impact.*

4.2.2.8 Public Private Partnerships

Key Assumptions

- The public and private sector would work together using their skills to improve access to HWWS opportunities.
- The private sector would help with the development of low cost and effective HWF.

Key Findings

- There was some buy in from the private sector primarily through the support of hand washing campaigns and soap sales.
- Private companies were engaged for specific events such as Global Hand Washing Day but not on a consistent day to day basis.
- The complete integration of public and private sector partnerships to achieve improved behaviour change communication and opportunities to wash hands with soap was not effectively realized in the 2011 – 2012 campaign.

Recommendations

- *The need for, and promotion of HWWS requires effective public private partnerships and these require to be engaged on a more regular and formal basis.*

4.3 The extent to which different programs, approaches and other cross cutting issues (by Govt, NGOs and developing partners) have contributed to the implementation of the ODF and HWWS strategies

Since the launch of the ODF Strategy and HWWS Campaign in 2011 and 2012, respectively, a number of sanitation and hygiene programmes have been implemented to support the Government of Malawi's efforts to meet its sanitation and hygiene goals both under the MDG and SDG frameworks.

4.3.1 Government Frameworks

To promote and ensure the support of Open Defaecation Free and Hand Washing With Soap by all by 2015, the Government of Malawi have a number of underpinning policies and strategies as outlined in Table 5.

Table 1: Policies and strategies that provide direct guidance for sanitation and hygiene in Malawi

Document	Key Focus
Malawi National Health Policy (2012)	Provision of health services, health promotion and disease prevention.
Health Sector Strategic Plan I and II (2011-2016)	Provision of health services, health promotion and disease prevention.
National Water Policy, 2007	Sustainable management and utilization of water resources, in order to provide water of acceptable quality and sufficient quantities, and ensure availability of efficient and effective water and sanitation services that satisfy the basic requirements of every Malawian and for the enhancement of the country's natural ecosystems.
The National Sanitation Policy (2008)	Promotion of participatory approach in sanitation and hygiene in rural, urban and institutional settings. The policy provides guidelines for the development of an Investment Strategy that will be supported by development Partners under a Sector Wide Approach (SWAp) for sanitation
National Open Defecation Free (ODF) Malawi Strategy 2011-2015	Elimination of OD in rural areas by 2015. CLTS the main tool for triggering, achieving and scaling up ODF
City Councils, Municipalities and district councils bye-laws	Promotion of sanitation and hygiene in the cities, municipalities and District Councils
The National Hand Washing Campaign. Strategy Malawi (2011 -2012)	Promotion of hand washing, integrating hand washing with the CLTS triggering
National Household Water Treatment and Safe Storage Action (2016- 2021) Plan	Reduction of faecal-oral diseases in Malawi by encouraging water treatment and safe storage using market based approaches
Malawi National Health Promotion Policy? (2013)	Provide guidance in the implementation of health promotion interventions for all the stakeholders in health and other sectors. 'The goal of the HP in Malawi is: ***'to reduce preventable deaths and disability through effective health promotion interventions'.***

Document	Key Focus
Infection control and waste management plan for Malawi, 2016	facilitate implementation of appropriate infection control and waste management practices across the three relevant sectors of Health, Mining and Labour, (which include work practice and administrative measures, environmental/engineering control, and use of appropriate personal respiratory protection, and improved waste collection, storage, treatment and disposal practices) to avoid infection and environmental pollution.
Guidelines for infection prevention and control for TB including MDR-TB and XDRTB	Infection prevention and control procedures to reduce the risk of M. tuberculosis transmission in health care facilities

Source: Malawi National Health policy, 2012. DeGabriele and Ngwale, 2017 & Stakeholders analysis, May 2017)

These Policies and Strategies provide the basis for supporting ODF and HWWS campaigns and programmes.

4.3.1 Supporting programmes

ODF and HWWS implementation have been supported by a number of programmes and agencies since 2011. Throughout this period 83 Traditional Authorities have been declared ODF which were funded primarily through the Global Sanitation Fund and UNICEF (Table 2)

Table 2: ODF Donors

Donor	Number of TAs declared ODF	Implementing partners
Global Sanitation Fund	28	United Purpose, Feed the Children, District Councils, DAPP, SOLDEV, Hygiene Village Project, World Relief
World Vision International	6	World Vision International, Water for People
UNICEF	35	United Purpose, Goal Malawi, World Vision International, Hygiene Village Project, CPAR, CADECOM, Care, PDI, District Councils
African Development Bank	2	Feed the Children, District Council
AusAid	6	Plan Malawi
Others	6	ICEDA, Water for People, Water Aid, Red Cross,

4.3.2.1 Global Sanitation Fund

One of the main programmes is the Accelerated Sanitation and Hygiene Practices Programme (ASHPP) (2010 – 2017) managed in country by Plan International and its

collaborating partners. The program was a government-led national initiative on sanitation and hygiene funded by the Water Supply and Sanitation Collaborative Council (WSSCC) through the Global Sanitation Fund (GSF). The programs aimed to support communities to achieve the following:

- Reduce open defecation, increase access to improved sanitation, coverage and use of safe hygiene practices
- Decrease in open defecation, increased use of improved sanitation and promote use of safe hygiene practices in rural districts with the lowest baseline and investment.
- Conduct sanitation and hygiene promotion campaigns.
- Develop capacity of local district government, civil society, and private sector involved in WASH.
  Support the planning and implementation of sanitation and hygiene promotion activities at district level (Plan international, June 2017).

Reports from the ASHP Program (2017 outcome survey Report) established an overall latrine coverage of 99.2% which was higher than 64.5% and 87% noted during baseline in 2012 and Outcome survey of 2014, respectively. Respondents were asked to show their latrine, there were no significant variations between males (98.8%) and females (99.5%). Pit latrine without slab /open pit was the most prevalent type of latrine owned by 82% of the respondent households who owned latrines. Most of the latrines had drop-hole covers (74.7%) this was contrary to the findings of the ODF sustainability studies conducted by DeGabriele and Ngwale (2017) and intact floors (84.4%) to assist in the reduction of flies moving in and out of the latrines. Overall the latrine utilization in all the surveyed areas was very high at 99.2%. This program as explained above had an objective of increasing sanitation to curb OD (Outcome Survey Final report, 2017).

Results from the ASHP Program (2017 outcome survey Report) further report that overall self-reported attainment of ODF status of communities of the respondents in the study conducted by Kumwenda et al. (2017) was at 82.9%. During the outcome survey, an assessment at household level was conducted to check if there was use of latrine by all household members and that there were no faeces in the household surrounding. Similarly, an observation was made to check presence/absence of faeces in the village. Inspections around the household premises found that most of the households (98.3%) did not have any human faeces in the household area or around the compound. However, about 37.8% of the households had animal faeces in the household area or around the compound. These results point that indeed other programmes contributed to the attainment of the ODF status in other areas.

4.3.2.2 UNICEF

UNICEF is a leading donor in the rural water supply and sanitation sector in Malawi. UNICEF is committed to supporting Government to develop capacities, policies and systems that will strengthen the sanitation sector as a whole. UNICEF's commitment to sector---wide initiatives were demonstrated through their support of CLTS programs in 14 districts across Malawi.

To this regard UNICEFs overall goal of the 2012 - 2016 in its Country (Malawi) programme is to support national efforts to progressively realise children's and women's rights through

improved child survival, development, protection and participation. The purpose of the survival component is to ensure that all children in Malawi reach their 5th birthday and thereafter grow and realize their full potential. Early childhood and adolescent development, basic quality education for all, participation by children and young people in matters of national interest, and prevention of HIV/AIDS among adolescents. Supporting the Government to develop a National Child Protection System. Moreover, UNICEF recognizes social protection as a fundamental right for children. Promoting social inclusion of the most marginalized households, especially children and women, through evidence-based advocacy with knowledge generation on emerging issues.

Ensuring that children and women have access to safe water and appropriate sanitation and that they learn healthy hygiene practices is a big determinant of children survival and development. UNICEF Malawi has taken slides in the promotion of Water, Sanitation and Hygiene with water-borne diseases being among the major causes of death in young children in Malawi, providing safe water and improved sanitation takes on urgent dimensions.

4.3.3 Model Districts

It is worth noting that Balaka district is an example district realized by stakeholders at both the learning event meeting of May, 2017 and also at the annual review meeting in November, 2017. Box 1 highlights the areas that contributed to their success

Box 2: Principles that facilitated the success of the ODF Initiative: The case of Balaka

Balaka district achieved over 95% Open Defecation Free (ODF) status five years after implementation of the CLTS intervention in its communities. Meanwhile, Balaka is known as the district that has more TA's declared ODF than any of the 28 districts in Malawi. The issues below, drawn from the positive lessons learnt in Balaka district, are key principles which enhanced the effective implementation of the ODF intervention:

Effective collaboration and better coordination: In order to identify, plan and develop options, a multi-sectoral approach was adopted. Partners were encouraged to work collaboratively together and in so doing provide a unified and seamless level of support to colleagues within and between institutions (e.g. to the DEHO, WASH, SHIN coordinators and NGOs at the district, EHO at the health centre to HSAs, volunteers and members at community level). This approach offered a joint initiative where members shared tasks to make the best use of skills and resources that were available both within and between institutions.

Participation by all: there was active participation by all players including holding planning meetings together (i.e. all partners) at district level, mapping out activities together, holding household visits together and evaluating the program together. In addition, the team encouraged information sharing at all levels.

Passion and Commitment: both implementers and community members were passionate and committed individuals and used these capabilities to work together to improve the CLTS intervention

Basic skills enhancement: although the ODF initiative did not require expert skills to implement since it only encourages an increase in latrine coverage (ref), still the awareness on ODF approach itself including construction of basic latrines and hand washing facilities needed some form of training. The following were done:

- Community workers were oriented and trained on CLTS
- Demonstrations on how to cast san-plats and hand washing facilities were done where necessary
- Communities were empowered to be able to make decisions about having latrines
- The district team conducted support visits to communities

Resources: the district had quite a good number of NGO's and this was indicated as one of the major reasons for the ODF success in Balaka. The NGO's were mindful that MoH cannot adequately finance the project and its associated activities. Recognition of this constraint assisted in the NGOs coming in to fund the main activities (e.g. field supervisions, trainings and orientation

4.4 Gaps in the ODF Malawi Strategy (2011 - 2015) and National Hand Washing with soap Campaign (2011 – 2012)

This section summarises the key gaps highlighted by stakeholders and outlined in literature.

4.4.1 ODF Strategy

As summarized in the review of the current strategy content there are a number of recommendations on content development and areas of the scope that require being included.

4.4.1.1 Widening of Scope

The gaps under widening scope include:

- Both the ODF and HWWS strategies do not provide specific definitions of latrines, hand washing facilities, etc. which leads to variation in implementation.
- The ODF strategy focuses only on the rural population, which has limited the focus and success of ODF achievement.
- ODF strategy implementation has focused on households with limited improvements in schools, and not action taken in other institutional settings. For example, declared areas have schools, prisons, markets and health facilities which do not have sufficient facilities and show evidence of open defaecation. During our survey, we found that most schools had basic latrines (i.e. with muddy floors and grass thatched) and often times the latrines were inadequate so that the pupils resorted to using the bush.
- There is no reference or integration of ODF strategy with menstrual hygiene management.
- Neither strategy has specific reference or support for vulnerable and marginalized groups.
- The ODF strategy does not consider the whole sanitation chain (capture to disposal).
- ODF strategy implementation was to be overseen by the National Open Defaecation Task Force (NOTF), which represents the Ministry of Health and the Ministry of Agriculture, Irrigation and Water Development with key development partners and civil society. This has not taken into consideration other ministries which are also crucial i.e. Ministry of Education, and others
- The current ODF strategy is limited to the use of CLTS and sanitation marketing and does not take into consideration the use of other participatory approaches such as PHAST and mechanisms to achieve ODF.
- Although there is the inclusion of 2 levels of ODF status, there is little reference to the effective use of the sanitation ladder to achieve continued improvement and sustainability.
- There is also a gap in harmonization of definitions such as the use of the word ODF. The JMP report defines OD as “Disposal of human faeces in fields, forests, bushes, open bodies of water, beaches or other open spaces, or with solid waste”. The percentage of households estimated to be OD is calculated by subtracting the percentage of households that have access to any facility. JMP has changed the definitions of the access levels: basic sanitation (i.e. use of what used to be called improved facilities that are not shared and use of higher service level which is called safely managed services); limited sanitation (meaning sharing of improved facilities) and unimproved (e.g. use of

facilities without a slab or platform). The definition and criteria used is different from that of Malawi Open defecation (OD) which is the disposal of human faeces into open spaces such as fields, forests, beaches or open water, including being mixed with solid waste and disposed of openly. The term is widely used in literature about Water, Sanitation, and Hygiene (WASH) issues in developing countries, including Malawi. Open Defecation Free (ODF), on the other hand, is when human faeces are safely disposed of, including into basic latrines or managed facilities. The difference is more on the calculation and verification (DeGabriele and Ngwale, 2017).

4.4.1.2 Sanitation and Hygiene Tools, infrastructure and Approaches

The tools and approaches used during ODF implementation and the following gaps were observed:

- The strategy did not include the standard for an acceptable toilet and hand washing structure. The strategy only stressed on increasing coverage.
- Suitable designs for different segments of communities e.g. the elderly, disabled, children and pregnant women were not considered. Our survey results shows that some pregnant women fail to use toilets as the entrance to the latrine is normally small to allow them get in. Provision for latrines suitable for the marginalized especially the physically challenged was not considered in the previous ODF 2011-2015 Strategy.
- There were no post ODF activities planned i.e. monitoring of the ODF declared areas and this led to slippage in ODF.
- Participants further observed that there was no emphasis on behaviour change, improvement of school sanitation, HWWS activities and no activities to equip children with behavior change information on HWWS.
- Some tools used for triggering communities were not acceptable in some cultures i.e. field work revealed that some Ngoni's in Mwanza did not like the issues of bringing faeces in public and most participants left while triggering was in session. However, during Male FGD, respondents preferred the other tools for triggering
- The development of the 2011 - 2015 strategy involved primarily CLTS stakeholders

4.4.1.2 Sanitation marketing

There was a clear knowledge gap in rural sanitation marketing (DeGabriele, 2009) notably:

- Designing lower cost latrines through reduced input of expensive materials such as cement
- Facilitating user choice on technology by presenting both construction costs, and operation and maintenance costs
- Providing access to sustainable credit services for construction
- Building capacity for affordable and sustainable support services such as toilet emptying, processing of waste, etc.
- Provision of an enabling environment for private enterprise

Stakeholders also stated that there is a need to have a better understanding of how sanitation and hygiene and directly or indirectly impact on household income.

4.4.1.3 Involvement of Leaders

As outlined above, the TA was recognized as a key level at which teams should be operating simultaneously to achieve accelerated implementation of CLTS and subsequent ODF status. It was reported that these team have been more effective in some TAs than others, and where leaders and TAs were not supportive of this programme there has been little progress. The gaps identified include:

- There was little integration of natural leaders into community structures i.e. village health committees. This was reported during stakeholder meeting.
- There was little reference from any stakeholder on the use or support of religious leaders in achieving ODF.
- Vulnerable and marginalized groups were supported in some areas, however it was felt that they should be engaged from the offset of the CLTS programme and be involved in the training, implementation and verification processes to ensure appropriate systems are in place to support them.
- Specific designs should be provided for suitable latrines for those with disabilities to ensure safe use and dignity.

4.4.1.4 Publicity

The majority of feedback regarding publicity pertained to the celebrations and ceremonies which take place when ODF is achieved at TA level. The successes and gaps found are outlined below:

- With regard to the awards given, results from FGDs and KII as well as stakeholders analysis revealed that giving was a good way of incentivizing the community. However, respondents noted that some Traditional leaders would keep the gifts (especially plastic basins and cups) for personal use.
- Although motivating, there is a high cost associated with the ODF celebrations that take place at TA level and the cost – benefit of these should be considered.
- Participants stressed the lack of progress after the ceremonies and celebrations have passed and the progressive slippage which then occurs back to OD.
- Integration of the ODF strategy with others such as HWWS was not achieved effectively.
- Integration of communication on ODF with other strategies was also limited.

4.4.1.5 Networking, Coordination and Integration

There was a gap in the understanding on what each actor is doing. This led to lack of proper coordination accelerated by fragmented work by the WASH actors in Malawi (WESNET DIRECTORY, 2017).

- Documentation of the success, failure, or lessons to be learned from CLTS and ODF achievements is haphazard. Naturally, there is an inclination for those involved in the innovation (either through its implementation or its funding) to claim success for it. There is however, little comparative documentation of what worked and what did not on a programmatic basis, to determine whether or not the projects were in fact replicated, or creatively adapted, for expansion (Dutton, et al, 2011) This review can help to identifying where and why they have failed or succeeded and the future route to be taken.

- Results from the FDGs and KIIs indicated that ODF achievement was attributed to support and zeal from partners, multi-sectoral collaboration and better coordination. One of the participants from the KIIs echoed that "*Proper coordination works wonders*".
- NOTF engaged partners well but governments have not improved funding of preventive WASH activities.
- The WASH sector in Malawi has vast actors but WESNET echoes that there was minimal coordination and knowledge sharing of efforts to drive a concerted agenda. There were very minimal efforts towards networking and learning in the WASH sub sector in Malawi.
- Fragmented work by the WASH actors in Malawi is perpetuated because of a gap in the understanding on what each actor is doing.

4.4.1 HWWS Campaign Strategy

During FDGs participants reported that people could not appreciate the importance of hand washing with soap as there is no visible link between non-washing of hands and diseases for people to appreciate and value the practice.

4.4.1.1 Development of Key Messages

Respondents indicated that the majority of households are aware of the key times when they should wash hands but there are still beliefs which affect the uptake of hand washing with soap.

- Households wash hands with water only and do not see the value of using soap to improve this.
- Child stools (particularly those from 0 – 6 months) do not contain bacteria which can cause illness.
- Hand washing facilities are not conducive to supporting effective and easy hand washing.
- Use of soap is prioritized for other household activities such as bathing, washing clothes, etc.
- When left at hand washing facilities soap is often stolen, or eaten by an animal which wastes a precious resource.

4.4.1.2 Integrating HWWS promotion in Community Led Total Sanitation Community Led Total Sanitation (CLTS).

- CLTS triggering focused on the construction and use of latrines with less emphasis on the need for HWWS.
- The use of disgust as a trigger for HWWS may not be an effective one and therefore not encourage practice.
- The ODF strategy only required the presence of a hand washing facility and soap to achieve Level 2 certification. To date communities have primarily achieved ODF at Level 1 which does not have a requirement for a hand washing facility and therefore there has been less emphasis on this practice.

4.4.1.3 Ensuring availability of hand washing facilities

- There is still a significant issue with the development and use of appropriate HWF in Malawi both at household at institutional level.
- Schools do not have suitable facilities to ensure effective HW or HWWS for all students.
- Households may have facilities but lack of access to water can mean they do not prioritise use for hand washing.
- HWF used at the moment are not durable or sustainable as they are damaged by animals and damaged by the sun after a few months exposure.
- Placement of soap at HWF is frowned upon as it is often stolen or eaten by animals.
- There is primarily an emphasis on HWWS for rural communities only without consideration to urban, institutional and workplace settings.

4.4.1.4 Use of social marketing and other Participatory approaches

- Social marketing of HWWS materials was minimal and based on small pilot testing of materials and systems with little learning shared in the WASH sector for potential scale up.
- The use of PHAST to promote hand washing was contradictory to the use of disgust through the CLTS approach. This may have led to confusion in facilitators on the approach to be used. This would also have been impacted by the greater emphasis on ODF achievement without HWWS.

4.4.1.5 Use of School Children as agents of change

- Little progress has been made in sanitation and hygiene practices in schools which are still underserviced.
- Current systems being put into schools for HW are not appropriate for the quick throughput of high numbers of students.
- Schools do not provide soap for students to use due to cost, theft, etc.
- Teachers are not integrating issues of HWWS into their day to day curriculum.
- There are currently no standard guidelines for sanitation and hygiene requirements at schools.
- Parents and other adults do not always value the opinion of children when they come home with hygiene messages *"you can do that at school but you do not need to do that at home"*

4.4.1.5 Use of Health Facilities models for hand washing with soap

- Most health facilities and other institutions in the ODF declared areas had no HWWS facilities.
- Health facilities and health workers were not promoting effective hygiene behaviours during clinics due to lack of facilities.

4.4.1.6 Use of Multiple Communication Channels for Behaviour Change

- Behaviour change messages were generalized and were not always based on sound research and understanding of how these would influence practice.
- Messages through different channels were not consistent and as such caused confusion.
- Institutional settings were not using good practice and were therefore not communicating effective behaviour change messages.

4.4.1.7 Public Private Partnerships

- There was some buy in from the private sector primarily through the support of hand washing campaigns and soap sales.
- Private companies were engaged for specific events such as Global Hand Washing Day but not on a consistent day to day basis.
- The complete integration of public and private sector partnerships to achieve improved behaviour change communication and opportunities to wash hands with soap was not effectively realized in the 2011 – 2012 campaign.

4.4.1.8 Other gaps identified

- The HWWS strategy is limited in terms of integration with other key strategies (e.g. Nutrition, mother and child health, immunisations, school health and nutrition, etc.) and relies largely on mass media rather than focused behavior change communication.
- Both strategies lack reference to specific standards and guides for the construction of toilets and hand washing facilities.
- HWWS strategy uses health facilities and schools as key locations for good practice and development of agents of change, but in many cases these were identified as having the poorest standards.

4.5 The extent that the changing assumptions and indicators impact achievement of ODF Malawi and hand washing behaviours

4.5.1 ODF and HWWS Changing Assumptions

As outlined in Section 4.2, there were a number of key assumptions made regarding the implementation of the ODF strategy and HWWS campaign between 2011 and 2015. These have been explored in detail in that section, however several assumptions have been identified which have directly impacted the achievement of ODF and HWWS in Malawi. These are summarized here and should be considered in conjunction with specific detail in Section 4.

4.5.1.1 CLTS as an effective tool to achieve ODF and HWWS

CLTS has taken hold across SSA since its introduction and is a very credible strategy for eliminating open defecation. Due to the speed with which it is being scaled up, its use as a primary vehicle of elimination of OD and the newness of the approach, it is essential to take note of key lessons which impact on effectiveness and sustainability.

At the time of the ODF strategy development for Malawi, the key stakeholders who participated were from organisations involved in CLTS piloting in Malawi. This system was being lauded and adopted across the world in LMIC countries to accelerate achievement of ODF, and to an extent has also achieved this in Malawi since 2011. Nevertheless, use of CLTS as the sole tool for achieving sustained ODF also has its limitations, and these must be carefully considered when moving forward to ODF Strategy 2018. As outlined previously, CLTS is not without , its limitations as a tool, and the impact expected from linked CLTS with sanitation marketing has not been realized to date in rural populations. However there were also concerns raised from respondents on the challenges of harmonisations across the country when so many partners are involved in implementation. During the stakeholders' analysis, there was a concern about moving towards harmonization, if that was interpreted to mean that one approach is the only approach. However it was agreed that there is significant value in trying to synchronize key principles that allow governments and implementers to make decisions that match the needs of their populations. There was also an agreement that other approaches should be considered and documented to support CLTS and that all approaches must emphasize use, equity and sustainability, and a shared commitment to working in partnership to avoid duplication, maximize resources and ensure impact. There was also the assumption that the HWWS campaign would work hand in hand with the CLTS triggering programme. However with a primary focus on achieving Level 1 ODF, there was little support or emphasis placed on the value and importance of HWWS.

4.5.1.2 HSAs and Traditional Leaders as Implementers

The strategy assumed the full and effective participation of HSAs and Leaders in the implementation of CLTS and achievement of ODF. There have been a number of challenges identified in this area which have hindered achievement of ODF.

As outlined previously, HSAs were active in many districts in implementation, and utilized their village health committees effectively to support this activity. However, particularly in those districts supported by partners, HSAs received additional payments and allowances to

undertake this work, and as such this did not effectively integrate the achievement and maintenance of ODF into their routine tasks. Once seen as a 'project' they were then unwilling to undertake sanitation related activities without the support of additional funds. It was agreed that HSAs are key to the achievement and maintenance of ODF, and they should be supporting and supervising their communities to achieve this as part of their routine activities. The production of the Community Health Strategy (2017 – 2022) and the Role Clarity Guidelines (2017) by the Community Health Services Section has helped to clarify structures for health service delivery at community level, and that includes the role of community health teams, HSAs and community health volunteers. These documents should be used as a guiding tool for the ODF strategy to ensure clarity and consistency for implementation.

In the case of leadership, there were a number of areas outlined including the use of traditional, religious and natural leaders. It is clear from this review that there have been varying degrees of success with the use of these leaders, and particular challenges with traditional leaders where they have failed to provide support. There have also been challenges where community health volunteers and leaders have not demonstrated good practice at their own households, and are therefore not the role models required to promote change. These situations have led to several concerns in ODF achievement. For example, leaders using by-laws to achieve ODF by refusing health services or extracting payment as fines. The strategy must consider whether some of these by-laws may lead to life threatening situations or go against human rights. Leaders may also favour friends and family during verification processes and protect those who are not meeting the target indicators thereby sending the wrong message to other community members, and leading to ODF certification where the necessary standards have not been met. It is therefore clear, that a more detailed understanding is needed of leadership roles in achieving ODF and supporting the process and sustained change of a community. There must be mechanisms that ensure transparency, objectivity and equity across all communities and households.

4.5.1.3 Verification of ODF would be a transparent objective process

The verification process is currently ineffective and costly. Inconsistent criteria and procedures for declaring, certifying and verifying ODF achievement have been reported as common constraints in many countries. In several countries where national criteria have not yet been agreed, different implementing agencies adopt different criteria and follow different processes, with some reported to be less rigorous than others, a situation which also exists in Malawi.

Indonesia and Timor-Leste were the only countries identified where an ODF verification process has been finalized at the national level. For example the stringent ODF criteria in Indonesia require that every household owns and uses an improved sanitation facility, whereas in other countries, including Timor-Leste, the ODF criteria allow some households to share latrines within ODF communities.

ODF verification is important because it provides some guarantee that commonly agreed ODF criteria have been reached, and that these criteria have been assessed by an independent group sometime after the ODF status was originally declared by the

community or implementing agency. While an ODF verification process will not tell us much about the sustainability of sanitation outcomes, it provides a more reliable source of progress data, and often encourages government involvement. Standardized ODF verification criteria are needed in order to assess the nation's progress towards meeting ODF and SDG 6, as well as to allow for progress comparison between districts. Maintenance of two levels of ODF achievement is recommended although the criteria within each should be reconsidered under consultation.

4.5.1.4 Maintaining ODF and climbing the sanitation ladder

The final key assumption made in the 2011 – 2015 strategy was that the achievement of ODF status would lead to sustained behavior change, and that households would begin to climb the sanitation ladder and begin to achieve Level 2 status. Instead, the current system has seen the celebration of ODF Level Status be followed by a lack of sustained change, and a level of slippage back to open defaecation which negates the initial achievement. This also removes any pressure or support to put in place infrastructure or change behaviour towards HWWS, as this was only an indicator of Level 2 ODF achievement.

The 2011 – 2015 strategy had a primary focus on 'triggering' communities into action; while considerably less resources and emphasis on following up and mentoring of communities 'post-triggering'. This was also identified as a concern by Thomas and Bevan (2014) who reviewed the processes and protocols for defining, reporting, declaring, certifying ODF and sustaining ODF, highlighting where the process varies between countries and potential determinants of sustainability within the process itself. They identified two key determinants to reduce slippage which were (1) quality of facilitation, and (2) post-triggering visits and monitoring. Quality of facilitation has varied across the country, and as stated above implementation has been undertaken by a number of different agencies. There was an assumption that achievement of ODF would indicate that communities had truly achieved ODF in terms of both infrastructure and behaviour change. However as evident from the section above, this has not been the case in many populations, with lip service paid to the process, and achievement being a result of peer pressure rather than a desire to change. This is compounded by the fact that many programs simply don't budget or have the timelines to support post-triggering follow on and see an ODF declaration as the chief outcome. However, in most cases, post certification is exactly the point at which communities are looking for support to access sanitation products and services and advice. Current programming and review processes must look at including innovations such as post-ODF sustainability plans and linking post-ODF monitoring more effectively with Sanitation Marketing efforts. The frequency of post-certification visits will vary depending on need, but should ideally be at least once per month during the first year.

This review process should also consider the standards by which a community achieves ODF status. At present Malawi has 2 levels (Box 1). It is recommended that these are subject to review, and that the strategy should reflect a wider scope for community hygiene standards than just the elimination of open defaecation. For example, the ODF protocol can be leveraged to yield enhanced health outcomes such as handwashing with soap and safe disposal of children's faeces which can easily be incorporated into the triggering process and which are key elements of the definition of maintaining an ODF environment. This could

also be extended in future strategies to the achievement of model or healthy communities with a wider range of integrated targets.

4.5.1.5 Using Institutions as model sites for education and support

Both the HWWS and ODF strategies required the use of schools, health facilities and hospitals as model sites for education and training on HWWS and promotion of ODF. All stakeholders indicated that the lack of specific requirements and targets for these facilities meant that these supposed 'model' institutions were in fact demonstrated poor practice rather than promoting the ideal. ODF and HWWS targets must include public spaces (e.g. markets) and institutions to ensure that standards are maintained across a population to ensure effective behaviour change is achieved and sustained.

4.5.1.6 Effective Behaviour Change Communication

Integral to the success of all these areas is the need for sustained behaviour change across the population. As such, there needs to be a well-trained quorum of community health teams volunteers who understand the key principles behind CLTS and HWWS behavior change practices. Only then will triggering, support and education be targeted effectively to achieve sustained behaviour change.

4.5.2 Cross cutting issues

If we are to achieve the SDG 6 targets by 2030, then the ODF and HWWS with soap strategies must consider emerging principles and recommendations, which work towards equitable and adequate sanitation for all by 2030. (Myers and Gnilo 2017). Key to this process is effective learning from the last 7 years. This is difficult as documentation of the success, failure, or lessons to be learned from these experiments are haphazard. Naturally, there is an inclination for those involved in the innovation (either through its implementation or its funding) to claim success for it. There is however, little comparative documentation of what worked and what did not on a programmatic basis, to determine whether or not the projects were in fact replicated, or creatively adapted, for expansion. By revisiting these sanitation programmes through this project and identifying where and why they have failed or succeeded, much can be learnt (Ministry of Health, Kenya, 2016)

4.5.2 General recommendations from stakeholders for inclusion to updated Strategy

Based on the findings of review, and the changing assumptions, there were a number of specific recommendations from stakeholders on required actions as summarized in Table 3.

Table 3: Summary of issues suggested being included in the new ODF and HWWS strategies

Focus	Action
**ODF certification**	Proper and strong criteria for declaring ODF. The following to be considered:<br>• Update ODF criteria to reflect SDGs and other country developments<br>• TAs that have a school, market or health facility without ODF should not be declared,<br>• Households to have all requirements (latrine, drop hole cover, hand washing facility, water and soap for washing hands etc.),<br>• Pay sudden visits in the communities to appreciate what is really happening.<br>• Proper guidelines for sampling and certification;<br>• Who to preside over if T/A attains ODF, the Minister need to come once to a district, that is if the whole district is ODF, otherwise for the T/As, NOTF can declare and the District Commissioner can preside over the celebrations
**Integration**	• ODF strategy should have clarity on how it will further integrate with other relevant policies i.e. health, education and nutrition.
**Funding**	• Need to increase the percentage of funds in the national budget that goes towards WASH related activities.<br>• Revisit the no subsidy notion on building of toilets in communities in relation to sustainability.
**Sustainability**	• Develop structured post ODF activities<br>• Specifically recognize natural leaders, supervise and support them<br>• Clearly define toilet standards to be used in CLTS – encourage the construction of durable facilities.<br>• Engage more extension workers (other than HSAs) in ODF strategies.
**Schools and institutions**	• SLTS guidelines to be developed and disseminated effectively.<br>• School toilet standards to be finalized and disseminated effectively.<br>• Ensure adequate toilets in schools. Furthermore, cleanliness of toilets in schools needs to be emphasized in order to encourage usage. Some students resort to using the bush due to inadequate toilets. However, some pupils don't use the few available toilets for lack of cleanliness.<br>• Minimum criteria for health facilities and public places to be developed and met.
**Innovation**	• Show a visible link between non washing of hands and diseases<br>• Develop more Behaviour Change techniques in the new strategies.<br>• Develop and introduce better sanitation technologies that can withstand bad weather<br>• Develop latrines suitable for the marginalized.<br>• Improve privacy in the toilets especially for women.<br>• Include the whole shit flow diagram in the new ODF strategies, what happens when the toilets are full, especially in schools? We may need to develop emptying strategies among others.
**Advocacy**	Wide dissemination of the new strategies at all levels (national to local).

4.6 How the strategies link in with other WASH related strategies and approaches

The now outdated ODF Malawi Strategy (2011 – 2015) and HWWS Campaign (2011-2012) was developed in line with one of the provisions within National Sanitation Policy 2006, which states that "Open defecation shall not be tolerated in Malawi". It was also developed in line with the National 10 Year Sanitation, Hygiene Investment Plan and the Sanitation and Hygiene Master Plan for Low income areas and the Health Sector Strategic Plan. The Sanitation Policy stresses the need to create public awareness on improved sanitation, create effective linkages between all relevant sanitation stakeholders and promote integrated and holistic planning, development and design of sanitation and hygiene promotions initiatives and programmes. The policy also emphasises the need for undertaking relevant training and capacity building of government staff, school children, teachers and community members in sanitation and hygiene promotion related issues.

At the time, the strategy and campaign were aimed at aligning, synchronizing and harmonizing sanitation and hygiene initiatives and interventions towards meeting the goals of the Malawi Growth and Development Strategy (MGDS) II (2012 – 2016) and the associated Millennium Development Goals (MDG) 1, 3, 4, 5, 6 and 7 by the year 2015. Prevention of diarrhoea and pneumonia would therefore contribute significantly in meeting Millennium Development Goal (MDG) number four which is aimed to reduce deaths in under five children by two thirds by 2015.

The outdated strategy needs to now be aligned with the core updated national policies with a specific focus on Sustainable Development Goals 6 which requires universal access to improved sanitation and hygiene by the year 2030. These include the Malawi Growth and Development Strategy III, Health Sector Strategic Plan II, Sanitation Policy and the Community Health Strategy (2017 – 2022). Any revisions and changes to the ODF and HWWS strategies will require integrating with the requirements of these focal documents.

4.6.2 Cross Cutting Programmes

In addition to the national strategies, there are other more specific interlinked programmes in the country which have worked with the ODF and HWWS programmes with varying success.

In relation to specific current programmes, stakeholders referred to links with the following:

- Trachoma programme which focuses on facial cleanliness, and is promoting hand washing. Currently the facial cleanliness is used for Infection prevention and implemented by AMREF, JHAPIEGO and Red Cross.
- Maternal and Neonatal Programmes through Water Aid.

Nevertheless, there are a significant number of other programmes where there needs to be improved integration and links between the ODF, HWWS and other strategies including (but not limited to):

- School Health and Nutrition,
- Extended Programme for Immunisation
- Nutrition
- Community Health
- Sexual and Reproductive Health
- Mother and Child Health
- Menstrual Hygiene

5.0 CONCLUSION

Overall, findings from the desk review, field research and stakeholder consultation meeting suggest that Malawi has made strides in increasing latrine coverage. Still there are challenges, weaknesses and gaps that need to be considered in revising the new ODF and HWWS strategies.

In relation to ODF and latrine coverage, the country has only managed to reach level one (1) of ODF where close to 80% of communities have been triggered and approximately 40% of TAs have been declared ODF. Specifically for the TAs declared ODF, there are no visible signs of human excreta signaling that community members dispose of their faecal matter in a pit-latrine. However, the available pit-latrines (i.e. traditional pit-latrines) are of poor quality, weak and cannot withstand extreme weathers (e.g. heavy rains and winds) and often end up collapsing. The country is yet to attain level 2 of ODF where more durable latrines (i.e. improved pit-latrines) should be constructed so as to sustain the ODF status. The evidence generated by this assignment supports the promotion of construction and use of improved pit latrines for scaling-up the latrine coverage in both households and institutions (e.g. schools) within the TAs. Apart from the poor quality of latrines in the TAs that were declared ODF, institutions (e.g. schools, hospitals, prisons, market places and trading centres) within the TAs have inadequate pit-latrine coverage. The evidence generated through this assignment suggests that policy makers and/or NOTF should ensure that for a TA to be declared ODF, any institutions within the TA should also demonstrate that it has adequate sanitation.

With regards to HWWS, a significant finding was that coverage of HWF is extremely low at around 30% and in selected TAs nationwide. A key challenge is the absence of durable HWF which can withstand extreme weathers (especially heat from the sun) and cannot be vandalized. This then calls for efforts to come up with hand washing facilities that are constructed not only using locally available materials but that they should be durable. The low coverage of HWF has also affected hand washing behavior where only a handful of community members reported that they wash their hands after using the toilet. Obviously with the low coverage of HWF, improper or ineffective handwashing is expected. Our review revealed that the practice of hand washing is a challenge even to households that have HWF. While some participants expressed that scarcity of water restricts them from having water in the hand washing facility and subsequently affecting their washing of hands after using the toilet, some participants expressed that their HWF are often vandalized by animals and children. From the review, it also emerged that HWWS is not commonly practiced and that soap is considered as a scarce and/or expensive and valued commodity that is usually prioritized for other important uses e.g. for washing clothes and not for hand washing. For many, washing their hands with plain water is enough. This finding illuminates a range of contextual factors that hinder hand washing and shows that HWWS is not primarily valued. This then calls for demonstrable innovations beyond the skill of making HWF that will make people change their behavior, value and prioritize HWWS.

It is clear from the feedback from all stakeholders and desk review, that future strategies must address concerns regarding integration of sanitation and hygiene programmes to ensure sustained change across Malawi and achievement of the SDGs by 2030.

With this in mind, it is the overall recommendation of this review that the current ODF and HWWS strategies should be integrated into a more general 'hygiene and sanitation' strategy. This would support not only the integration of HWWS and ODF programmes, but also the inclusion of key issues raised in stakeholder meetings such as menstrual hygiene management and solid waste management (including faecal sludge management). This would be an all encompassing strategy which targets rural and urban populations, domestic houses, commercial premises and institutions across the country. Only then can Malawi truly meet the target of Universal Sanitation and Hygiene for All.

REFERENCES

Cairncross, S., Hunt , C., Boisson, S., Bostoen, K., Curtis,V., Fung, CH & Schmidt WP (2010), Water, sanitation and hygiene for the prevention of diarrhoea. International Journal of Epidemiology 2010; 39:i193–i205

Curtis V, Cairncross S. (2003). Effect of washing hands with soap on diarrhoea risk in the community: a systematic review. Lancet Infect Dis 2003; 3:275–81.

Chambers, R. 2009. Going to scale with CLTS: Reflections of experience, issues and ways forward. *Institute of Development Studies, University of Sussex, United Kingdom.*

DeGabriele, J & Ngwale, M. (2017), ODF Slippage Report.

DeGabriele J. 2009 Sanitation Sector Status and Gap analysis: Malawi. Prepared for the *Global Sanitation Fund and the Water Supply and Sanitation Collaborative Council*.

Dutton, P.,Blackett, I., Nguyen, N., Rafiqah, I., Sarasdyan, W., Nguyen, M., Grossman, A & Weitz, A. (2011)Water and Sanitation Program, (2011) Handwashing with Soap- Two paths to National Scale Programs: Lessons learned from the field: Vietnam and Indonesia, World Bank

Kafanikhale, H (2017). Legal Instruments and policy frameworks for sanitation and Hygiene in Malawi. Presentation at the Annual Review meeting on ODF

Freeman, MF. Stocks, ME., Cumming, O., Jeandron, A., Higgins, PT., Wolf J., Pr€uss-Ust€un, A., Bonjour,Hunter8,PR Fewtrell, M–& Curtis, V ((2014). Hygiene and health: systematic review of handwashing practices worldwide and update of health effects. Tropical Medicine and International Health. Vol 19(8); pp 906-916

Kumwenda, S, Chidziwisano, K., Kalumbi, L., (2017). Outcome Survey Report: Accelerating Sanitation and Hygiene Practices Program (ASHPP), Plan International, Malawi

Kov Phyrum, Pedi Danielle Christina, Smets Sussanna (2012). Cambodia – Sanitation marketing lessons from Cambodia: a market-based approach to delivering sanitation. Water and Sanitation Program field note. Washington, DC: World Bank.

Luby et al (2005). Effect of handwashing on child Health. A randomized controlled trial. The Lancet

Malawi WESNET directory, 2017. wesnet

Ministry of Health, Kenya (2016). **National ODF Kenya 2020 Campaign Framework** 2016/17. Ministry of Health

Ministry of Health.(2015). Malawi ODF 2011-2015 strategy, Ministry of Health, Malawi

Ministry of Agriculture, Irrigation and Water Development, (2008), Malawi National Sanitation Policy (2008). Ministry of Agriculture, Irrigation and Water Development

Myers and Gnilo (2017) Supporting the Poorest and Most Vulnerable in CLTS Programmes The CLTS Knowledge Hub Institute of Development Studies at the University of Sussex, Brighton

ODF, Rural Kenya. (2013). Verification and Certification of ODF communities in Kenya, Ministry of Health, Kenya

Strunz EC, Addiss DG, Stocks ME, Ogden S, Utzinger J, Freeman MC (2014) Water, Sanitation, Hygiene, and Soil-Transmitted Helminth Infection: A Systematic Review and Meta-Analysis. PLoS Med 11(3): e1001620. https://doi.org/10.1371/journal.pmed.1001620

UNICEF (2013). Community-led total sanitation in East Asia and Pacific; Progress, Lessons and Directions, UNICEF - East Asia and Pacific Regional Office

UNICEF / WHO Joint Monitoring Program (JMP). 2017, Progress on Sanitation and Drinking water 2017 update and MDG Assessment, UNICEF / WHO Geneva

Thomas, A and Bevan, J. (2014) Developing and Monitoring Protocol for the Elimination of Open Defecation in Sub-Saharan Africa

World Bank (2018) Innovations in WASH Impact Measures: *Water and Sanitation Measurement Technologies and Practices to Inform the Sustainable Development Goals.* ISBN (electronic): 978-1-4648-1198-2
